# Trends in stroke incidence, death, and disability outcomes in a multi-ethnic population: Auckland regional community stroke studies (1981–2022)

**DOI:** 10.1016/j.lanwpc.2025.101508

**Published:** 2025-03-10

**Authors:** Valery L. Feigin, Rita Krishnamurthi, Balakrishnan Nair, Ilari Rautalin, Varsha Parag, Craig S. Anderson, Bruce Arroll, P. Alan Barber, Suzanne Barker-Collo, Derrick Bennett, Paul Brown, Dominque A. Cadilhac, Jeroen Douwes, Daniel Exeter, Anna Ranta, Yogini Ratnasabapathy, Andrew Swain, El-Shadan Tautolo, Braden Te Ao, Amanda Thrift, Bronwyn Tunnage

**Affiliations:** a National Institute for Stroke and Applied Neurosciences, Faculty of Health and Environmental Sciences, AUT University, Private Bag 92006, Auckland, New Zealand; b University of Helsinki, Finland; c National Institute for Health Innovation, The University of Auckland, Auckland, New Zealand; d The George Institute for Global Health, New South Wales, Australia; e University of New South Wales, Australia; f Neurology Department, Royal Prince Alfred Hospital, Sydney, Australia; g Institute for Science and Technology for Brain-inspired Intelligence, Fudan University, Shanghai, China; h Faculty of Medical and Health Sciences, General Practice and Primary Healthcare, The University of Auckland, New Zealand; i University Research Centre for Brain Research, The University of Auckland, New Zealand; j School of Psychology, The University of Auckland, Auckland, New Zealand; k Nuffield Department of Population Health, University of Oxford, Oxford, UK; l University of California, Merced, CA, USA; m Stroke and Ageing Research, Department of Medicine, School of Clinical Sciences, Monash University, Clayton, Victoria, Australia; n Centre for Public Health Research, Massey University, Wellington, New Zealand; o Faculty of Medical and Health Sciences, The University of Auckland, New Zealand; p Department of Medicine, University of Otago, Wellington, New Zealand; q Department of Neurology – Wellington Hospital, New Zealand; r Te Whatu Ora, Health New Zealand – Waitematā, New Zealand; s Research and Education, Kia Ora te Tangata - Wellington Free Ambulance, Wellington, New Zealand; t School of Clinical Sciences, Auckland University of Technology, Auckland, New Zealand; u AUT Pacific Health Research Centre, Auckland University of Technology, Auckland, New Zealand; v School of Population Health, The University of Auckland, New Zealand; w Faculty of Medicine, Nursing and Health Sciences, Monash University, Clayton, Victoria, Australia

**Keywords:** Stroke, Trends, Outcomes, Incidence, Mortality, Disability

## Abstract

**Background:**

Reliable data on trends of stroke incidence and outcomes over time are necessary for assessing the effectiveness of public health and clinical strategies, and for allocating healthcare resources. We assessed the levels and trends in incidence, mortality, early case fatality and disability for stroke in a defined, ethnically mixed population over 40 years.

**Methods:**

To analyse data from five population-based stroke incidence studies in adult residents (age ≥15 years) of the Greater Auckland Region of New Zealand (NZ) (1.35 million) over 12-month calendar periods for 1981–1982, 1991–1992, 2002–2003, 2011–2012, and 2021–2022. Fatal and non-fatal, hospitalised and non-hospitalised stroke events (first-ever and recurrent) were identified through multiple overlapping sources using clinical World Health Organization (WHO) diagnostic criteria and neuroimaging to define three major pathological types of stroke: ischaemic stroke (IS), primary intracerebral haemorrhage (PICH), subarachnoid haemorrhage (SAH), and stroke of undetermined type (SUT). Crude and age-standardised annual incidence, mortality, 28-day case fatality and disability level, and 40-year trends were calculated by age, sex, and ethnicity assuming a Poisson distribution. For comparison of our findings, we carried out a pooled analysis of methodologically comparable population-based stroke epidemiology estimates in high-income countries over the last two decades.

**Findings:**

Overall, there were 7462 first-ever strokes (9917 events) over the 40-year period (4,682,012 person-years). From 1981–1982 to 2021–2022, age-standardised stroke incidence rates decreased from 156/100,000 (95% confidence interval [CI] 143; 170) to 124/100,000 (119; 130) and mortality rates from 98/100,000 (88; 110) to 28/100,000 (26; 31) in nearly all age, sex, and ethnic groups. Moreover, from 2002–2003 to 2021–2022, there was an increase in stroke incidence of 1.28% per year (95% CI 0.38–2.17) in people aged 15–54 years, with the mean age of people with stroke decreasing from 73.0 (SD ± 13.8) in 2002–2003 to 71.6 (SD ± 14.9) in 2011–2012 and 70.7 (SD ± 15.2) years in 2021–2022 (p for trend <0.0001). The risk of stroke in Māori and Pacific people in 2021–2022 was almost 1.5 and 2.0 times greater than that in NZ Europeans. Ethnic disparities in the risk of stroke and age of stroke onset remained stable over the study period. From 1981–1982 to 2021–2022, 28-day stroke case fatality declined from 33.1% to 12.1% (p < 0.0001). There was a trend towards reducing 28-day case-fatality (from 31.6% [95% CI 27.6; 35.7] in 1981–1982 to 11.4% [10.0; 12.7] in 2021–2022) and an increasing proportion of stroke survivors with good functional outcome at discharge/28-days post-stroke (increased from 45.7% (95% CI 41.3; 50.0) in 1981–1982 to 60.2% (58.1; 62.3) in 2021–2022).

**Interpretation:**

Stroke incidence, 1-year mortality and 28-day case-fatality and disability have decreased in Auckland, NZ over the last 4 decades. However, over the last decade (2011–2022) there was a stagnation in the decline in the age-standardised stroke incidence rates. The absolute numbers of people with strokes, and those who have died or remained disabled from stroke, have significantly increased from 1981 to 2022. Ethnic disparities in the risk and burden of stroke persist. Effective prevention strategies for stroke must remain a high priority.

**Funding:**

10.13039/501100001505Health Research Council of New Zealand.

**Research in context**

**Evidence before this study**

Previous research shows a consistent decline in age-standardised stroke incidence and mortality rates globally in the last half of the 20th century. There has been a subsequent deceleration in this decline, and an overall flattening of the decline in the past few years. Since 2010, age-standardised stroke mortality rates have increased in some locations (e.g., China, Indonesia, some parts of the United States, Mexico, United Kingdom), and there has been a significant increase in the incidence of stroke in younger individuals aged <55 years in various high-income countries. There is also evidence suggesting that in some high-income countries, stroke severity has decreased over the last 20 years, and favourable functional outcomes have increased in some subgroups of individuals with stroke. However, such evidence should be confirmed in other populations, and there remains no reliable population-based data on long-term trends in case-fatality and functional outcomes after stroke. Our last report on trends in stroke incidence, mortality and case-fatality in the Greater Auckland Region covered the period from 1981–1982 to 2011–2012 and did not include an analysis of stroke disability. To identify comparable epidemiological studies and analyse them for contemporary trends in stroke incidence and outcomes we searched Medline, Scopus, Google Scholar, and PubMed with the terms “stroke”, “cerebrovascular events/disease”, “registry”, “survey”, “epidemiology”, “incidence”, “case-fatality”, “mortality”, “morbidity”, “ethnic/racial”, “disability”, “trend(s)”, and “population or community based” carried out between 1 January 2000 and 31 December 2024 and published in English.

**Added value of this study**

This large mixed method population-based study for the first time provides reliable estimates on the current level and 40-year trends in stroke incidence (incident and recurrent strokes), mortality, 28-day case-fatality and disability in a multi-ethnic population. For comparison of our findings, we also carried out a pooled analysis of population-based estimates in high-income countries over the last two decades. We found that although overall stroke incidence and mortality rates declined from 1981–1982 to 2021–2022, there has been a stagnation of reduction of incidence rates over the last decade, particularly in Māori and Pacific people of younger age. There was also a deepening of ethnic disparities between New Zealand Europeans and Māori, Pacific and Asian/other people, and a significant increase in the absolute number of people who experienced a new stroke, died from, survived and remained disabled after a stroke.

**Implications of all the available evidence**

These findings re-enforce the importance of evidence-based stroke care, prevention, and rehabilitation for stroke in New Zealand and across the world. They also confirm previous observations of an increasing burden of stroke, especially in younger people and subpopulations. The development and implementation of new, population-wide and targeted means of stroke prevention remains a high priority.

## Introduction

The global epidemiology and natural history of stroke are changing fast with changing population age structures, socio-demographics, risk factor profiles, healthcare services, treatments and preventative strategies.^1^ Accurate data on secular trends in stroke morbidity, mortality, case-fatality, and disability are of crucial importance for assessing the effectiveness of strategies used for primary and secondary stroke prevention, stroke health care delivery, evidence-based health care planning and resource allocation. Although overall global age-standardised stroke incidence and mortality rates have declined since 1990,^1^ there has been a recent levelling off in some of these positive trends.^1^ Moreover, age-standardised mortality rates have increased in some locations (e.g., China, Indonesia, some parts of the United States, Mexico, United Kingdom)^2^ while the age-standardised stroke incidence rates have increased in younger individuals (under 55 years of age).^3^, ^4^, ^5^ These trends have resulted in a significant increase over the last three decades in the absolute number of people affected by stroke, people who died due to stroke or people who remained disabled after stroke, especially in low-to middle-income countries (LMICs) but also in some high-income countries (HICs).^1^ While there is some evidence that stroke severity has decreased over the last 20 years in some HICs,^6^^,^^7^ and favourable functional outcomes have increased in some subgroups with stroke,^6^, ^7^, ^8^ the rate of recurrent strokes over a similar period of time has not substantially reduced in many countries,^9^ except for Sweden and The Netherlands.^10^^,^^11^ In addition, racial/ethnic disparities in stroke frequency and outcomes persist over time.^12^, ^13^, ^14^, ^15^, ^16^, ^17^ The factors responsible for these unfavourable and often discordant trends are not fully understood, largely because of the lack of reliable population-based stroke surveillance studies. Even less data are available on secular trends in stroke burden and disability outcomes in different ethnic groups.

The most reliable way to address these questions is to conduct a series of large, prospectively collected and methodologically comparable stroke frequency and outcome studies that meet the criteria for an ‘ideal’ population-based study^18^ in an ethnically and socio-demographically diverse population. To cover different periods of the practical use of various preventative, treatment and health care delivery strategies, these studies should also preferably be conducted over several decades. However, because such studies are complex,^19^^,^^20^ high-quality population-based data on secular trends in stroke incidence, mortality, and functional outcomes, specific to stroke pathological and aetiological type, age, sex, and ethnicity are lacking. To address this gap, we used ‘ideal’ population-based data from the five Auckland Regional Community Stroke studies (ARCOS I–V) (1981–1982, 1991–1992, 2002–2003, 2011–2012, and 2021–2022) to determine 40-year trends (1981–2022) in first-ever and recurrent stroke occurrence, 28-day case-fatality, mortality and disability outcomes in four major ethnic groups (NZ European, Asian/other, Māori, and Pacific people) by age, sex, and three pathological types of stroke in Auckland, New Zealand. For comparison of our findings, we also carried out a pooled analysis of methodologically comparable population-based stroke epidemiology estimates in HICs over the last two decades.

## Methods

### Study methodology

The study population and methods of case ascertainment in the five ARCOS studies are described in detail elsewhere.^21^, ^22^, ^23^, ^24^, ^25^ In brief, these studies comprised harmonised population-based identification methods and registers of all new cases of stroke (fatal and non-fatal, hospitalised and non-hospitalised) in the greater Auckland region over consistent 12-month calendar periods. The total resident population of the catchment area aged ≥15 years grew from 596,580 in 1981–1982 to 1,346,900 in 2021–2022. All five studies incorporated the use of WHO clinical diagnostic criteria^26^ for stroke and multiple overlapping methods of prospective case ascertainment, including systematic searches of hospital admissions, discharge registries, computed tomography (CT)/magnetic resonance imaging (MRI) records, general practitioner (GP) records, private hospitals, rest homes, rehabilitation services, NZ Health Information Service data of all fatal and non-fatal stroke and transient ischaemic attack (TIA) events, national death certificates, and coroner/autopsy records occurring within and just outside of the study population area. While for the first two ARCOS studies (1981–1982 and 1991–1992) a cluster sample of 50% and 25% of GP records were used to estimate the total number of non-hospitalised, non-fatal stroke events,^16^ the last three ARCOS studies (2002–2003, 2011–2012, and 2021–2022) systematically monitored (daily, weekly or monthly) the whole study population of the catchment area for new stroke events. We report on pathological types of stroke (ischaemic stroke [IS], primary intracerebral haemorrhage [PICH], subarachnoid haemorrhage [SAH], and stroke of undetermined pathological type [SUT]) only for studies where no less then 70% of acute incident stroke were verified by CT/MMI/autopsy. Therefore, data on IS and PICH were not presented for the 1981–1982 and 1991–1992 studies, although SAH was verified in these studies by either lumbar puncture or autopsy, and, therefore, included in the analysis of SAH trends. For additional details on study methodology, please see Appendix pp. 1–2.

To enable valid comparisons, all new stroke cases, including suspected strokes and TIAs, were ascertained by stroke physicians of the study Stroke Adjudication Committee. Similar to the methodology used in the ARCOS III and ARCOS IV studies, stroke risk factors, management variations and medication-use in the ARCOS V study were ascertained via hospital and outpatient medical records. Baseline variables obtained from medical records included socio-demographics, stroke, medical and family history, risk factors, results of routine cardiovascular and neurological examinations, pre-morbid disability, level of dependency at discharge and a functional and self-care assessment. Disability level during 28 days after stroke onset (as measured by modified Rankin Scale [mRS] in individuals with first-ever stroke only)^27^ was based on in-hospital assessment, discharge summary, or by a telephone assessment if the patient had been discharged home or into residential care. All study participants were followed up at 1 year for fatal/non-fatal outcomes. We used the national register of deaths to determine the survival status of all individuals with a new stroke across all five studies from 1981 to 2024. Ethnicity was identified by self-report across all five ARCOS studies and prioritised as a single ethnic group for the purposes of analysis in the following order: Māori, Pacific, Asian/other, NZ European.

### Statistical analyses

Descriptive statistics were used to assess baseline socio-demographic and medical characteristics of people with stroke by ethnic group across the 40-year study period. Statistical significance of changes in the distribution of categorical variables was tested using the Cochrane–Armitage method^28^ and for continuous variables using the Kruskal–Wallis nonparametric ANOVA. Crude annual age-, sex-, and ethnic-specific incidence (first-ever-in-a-lifetime events) and attack rates (all events including first-ever and recurrent) of stroke per 100,000 population per year with 95% confidence intervals (CI), were calculated assuming a Poisson distribution. Age, sex, and ethnic structures of the corresponding Auckland census data were used as the denominators in calculating incidence and mortality rates. Age-standardised rates were derived by the direct method using the WHO world standard population as the reference. The Cochrane–Armitage test for trends^28^ was used to analyse 40-year changes in stroke incidence, 28-day case-fatality (proportion [%] of people with stroke who died within 28 days of stroke onset among the total number of people with incident stroke), mortality rates (number of people with incident stroke who died in the study population over the one-year follow-up period [numerator] divided by the study population at risk [denominator]), and disability outcome at discharge/28 days as measured by the mRS dichotomised into two categories—good functional outcome (mRS score of 0–2) and poor functional outcome (mRS score of 3–5). In the 1981–1982 study only motor deficit was evaluated - no/mild motor deficit was considered a good functional outcome, while moderate/severe motor deficit was considered as poor functional outcome. P-values were 2-sided and the conventional 5% level was considered to be statistically significant.

For ethnic comparisons, rate ratios (RRs) of age-standardised rates were calculated using the NZ/European population as the reference. RRs were also calculated to evaluate differences in age-standardised rates between the 1981–1982 (reference period) and 2021–2022 study periods, and the Wald statistic tests of heterogeneity across age groups were performed.^29^ Similar to our previous report,^16^ completeness of case ascertainment based on the sources of notification was determined using capture-recapture techniques.^30^

To pool the trends in first-ever stroke incidence, case-fatality and dependency rates between the ARCOS III and V studies with other comparable population-based stroke incidence and outcome studies during the early 21st century, and assuming that findings could vary between studies due to differences among studies, we conducted random-effects meta-analyses.^31^ Details of the literature search for that analysis are presented in Appendix pp. 3–4. Due to the variation of time intervals, we calculated average annual percentage changes (AAPC) with 95% CIs between the mid-years of the first and the last periods. Pooled incidence trend estimates were calculated separately for young (<45 or <55 years of age) and older (≥45 or ≥55 years of age) individuals, while trend estimates for 28-day case-fatality (within 28 or 30 days after stroke) and disability (disabled [mRS 3–5] vs non-disabled [mRS 0–2]) were calculated for all ages. Moreover, we used the I^2^-test to assess the between-cohort heterogeneity, and categorised heterogeneity according to the Cochrane Collaboration recommendations.^32^ Pooled analyses were only done when three or more studies met the inclusion criteria. SAS 9.4 was used for all the analyses.

Each study was approved by the Health and Disability Ethics Committee of New Zealand and the last two studies (2011–2012 and 2021–2022) were also approved by the Auckland University of Technology Ethics Committee.

### Role of the funding source

The funder of the study had no role in study design, data collection, data analysis, data interpretation, or the writing of this article.

## Results

### Demographic characteristics, risk factors profile, and acute management

Using 4,682,012 person-years of observation, there were 7462 first-ever-in-a-lifetime stroke events registered across the five studies (Table 1; for more details see Appendix pp. 3–4). From 1981–1982 to 2021–2022, there was a significant increase in hospitalisations of people with stroke, brain CT/MRI within the first 7 days of hospital admission, and treatment in acute stroke units. Over the 40-year study period among individuals with a stroke, we observed a significant increase in the prevalence of pre-stroke hypertension, myocardial infarction, type 2 diabetes mellitus, use of blood pressure and lipid-lowering medications, antiplatelet agents, and anticoagulants. Over the last 20 years, the prevalence of AF in individuals with stroke was significantly reduced by about 2% (although it was increased in 2011–2012), but only because of the corresponding reduction in the prevalence of AF in people of Asian/other ethnicity, with no change in other ethnic groups. The prevalence of current smoking in individuals with stroke decreased substantially for the overall period (from 1981–1982 to 2011–2012), but increased significantly from 2011–2012 to 2021–2022 across all ethnic groups except for Māori. In Māori, the prevalence of smoking remained very high across all study time periods and had even increased over the last two decades (38.9% in 2002–2003 and 46.9% in 2021–2022).

**Table 1:** Baseline characteristics, management and 28-day outcomes of stroke events in each ARCOS study over the last 40 years (1981–1982, 1991–1992, 2002–2003, 2011–2012, and 2021–2022) by ethnicity.

	1981–1982 n (%)	Missing cases (n)	1991–1992 n (%)	Missing cases (n)	2002–2003 n (%)	Missing cases (n)	2011–2012 n (%)	Missing cases (n)	2021–2022 n (%)	Missing cases (n)	P for trend^a^
**Total number of participants**	1360	0	1761	0	1938	0	2096	0	2515	0	
**Demographics**											
Male	662 (48.7)	0	817 (46.4)	0	892 (46.0)	0	1012 (48.3)	0	1312 (52.2)	0	0.002
Age, mean (SD), years											
NZ/European	72.2 (12.8)	0	73.5 (12.1)	0	75.6 (12.5)	0	75.3 (13.4)	0	75.4 (13.4)	0	<0.0001
Māori	56.7 (14.2)	0	55.0 (16.1)	0	60.7 (14.3)	0	59.6 (15.5)	0	60.9 (14.4)	0	0.016
Pacific	55.8 (9.0)	0	59.7 (15.0)	0	64.5 (13.6)	0	61.6 (14.9)	0	62.1 (14.7)	0	0.006
Asian/other	72.1 (12.8)	0	65.6 (13.2)	0	65.9 (13.9)	0	67.5 (13.3)	0	67.9 (15.3)	0	0.283
Overall	71.2 (13.3)	0	71.6 (13.5)	0	73.0 (13.8)	0	71.6 (14.9)	0	70.7 (15.2)	0	<0.0001
Ethnicity											<0.0001
NZ/European	1248 (91.8)	0	1532 (87.0)	0	1431 (75.6)	0	1434 (68.5)	0	1438 (57.2)	0	
Māori	60 (4.4)	0	82 (4.7)	0	102 (5.4)	0	138 (6.6)	0	212 (8.4)	0	
Pacific	32 (2.4)	0	111 (6.3)	0	197 (10.4)	0	270 (12.9)	0	367 (14.6)	0	
Asian/other	20 (1.5)	0	36 (2.0)	0	162 (8.6)	0	252 (12.0)	0	498 (19.8)	0	
Missing	0	0	0	0	46	0	2	0	0	0	
**Source of notification**											<0.0001
Hospital	1082 (80.0)	0	1092 (62.0)	0	1361 (70.2)	0	1733 (82.7)	0	2469 (98.2)	0	
General practitioner	30 (2.2)	0	368 (20.9)	0	117 (6.0)	0	2 (0.1)	0	5 (0.2)	0	
Death certificate	104 (7.7)	0	161 (9.1)	0	95 (4.9)	0	15 (0.7)	0	8 (0.3)	0	
Other sources	136 (10.1)	0	140 (8.0)	0	365 (18.8)	0	346 (16.5)	0	33 (1.3)	0	
Missing	8	0	0	0	0	0	0	0	0	0	
**Premorbid risk factors (from medical notes)**											
High blood pressure											
NZ/European	632 (51.1)	0	802 (52.7)	9	783 (57.7)	74	947 (66.0)	0	810 (56.3)	0	<0.0001
Māori	38 (63.3)	0	41 (52.6)	4	63 (62.4)	1	85 (61.6)	0	96 (45.3)	0	0.0154
Pacific	22 (68.8)	0	49 (45.0)	2	124 (65.6)	8	178 (65.9)	0	241 (65.7)	0	0.0213
Asian/other	8 (40.0)	0	18 (50.0)	0	88 (58.7)	12	184 (73.0)	0	292 (58.6)	0	0.469
Overall	700 (51.5)	0	910 (52.1)	15	1079 (59.0)	109	1394 (66.5)	0	1439 (57.2)	0	<0.0001
Myocardial infarction											
NZ/European	146 (11.8)	10	273 (17.9)	10	190 (13.5)	28	401 (28.0)	0	292 (20.3)	0	<0.0001
Māori	8 (13.8)	2	9 (11.4)	3	12 (11.9)	1	25 (18.1)	0	41 (19.3)	0	0.059
Pacific	2 (6.7)	2	3 (2.8)	2	15 (7.9)	6	34 (12.6)	0	48 (13.1)	0	0.001
Asian/other	0	2	3 (8.3)	0	17 (11.0)	8	47 (18.7)	0	74 (14.9)	0	0.077
Overall	156 (11.5)	16	288 (16.5)	15	240 (12.7)	50	507 (24.2)	0	455 (18.1)	0	<0.0001
Previous stroke											
NZ/European	314 (25.3)	0	404 (26.4)	0	361 (25.5)	18	308 (21.5)	2	258 (17.9)	0	<0.0001
Māori	14 (23.3)	0	21 (25.6)	0	12 (11.9)	1	21 (15.2)	0	41 (19.3)	0	0.341
Pacific	2 (6.3)	0	25 (22.5)	0	54 (27.8)	3	62 (23.2)	3	71 (19.3)	0	0.634
Asian/other	0	0	6 (16.7)	0	36 (23.1)	6	56 (22.3)	0	84 (16.9)	0	0.755
Overall	330 (24.3)	0	456 (25.9)	0	477 (25.1)	38	448 (21.4)	5	454 (18.1)	0	<0.0001
Diabetes mellitus											
NZ/European	98 (7.9)	8	193 (12.6)	6	179 (12.7)	20	236 (16.5)	0	236 (16.4)	0	<0.0001
Māori	20 (33.3)	0	19 (24.4)	4	35 (34.7)	1	41 (29.7)	0	62 (29.2)	0	0.822
Pacific	12 (46.2)	6	16 (15.0)	4	69 (36.1)	6	117 (43.3)	0	194 (52.9)	0	<0.0001
Asian/other	4 (20.0)	0	8 (22.2)	0	40 (26.1)	9	77 (30.6)	0	175 (35.1)	0	0.005
Overall	134 (10.0)	14	236 (13.6)	14	329 (17.4)	43	471 (22.5)	0	667 (26.5)	0	<0.0001
Atrial fibrillation											
NZ/European	NA	0	NA	0	328 (23.7)	46	460 (32.1)	0	336 (23.4)	0	0.798
Māori	NA	0	NA	0	29 (28.7)	1	42 (30.4)	0	51 (24.1)	0	0.285
Pacific	NA	0	NA	0	41 (21.5)	6	62 (23.0)	0	71 (19.3)	0	0.452
Asian/other	NA	0	NA	0	18 (11.9)	11	47 (18.7)	0	43 (8.6)	0	0.024
Overall	NA	0	NA	0	416 (22.0)	74	611 (29.2)	0	501 (19.9)	0	0.0039
Current smoking											
NZ/European	330 (26.7)	12	330 (21.7)	8	162 (12.6)	141	178 (12.8)	44	482 (34.1)	26	<0.0001
Māori	32 (53.3)	0	41 (50.6)	1	35 (38.9)	12	55 (40.4)	2	98 (46.9)	3	0.241
Pacific	12 (37.5)	0	31 (28.7)	3	23 (13.1)	21	65 (24.4)	4	120 (33.2)	6	<0.0001
Asian/other	0	0	9 (25.0)	0	15 (10.3)	17	23 (9.3)	6	105 (21.5)	10	<0.0001
Overall	374 (27.7)	12	411 (23.5)	12	241 (14.0)	219	322 (15.8)	57	805 (32.6)	45	<0.0001
**Premorbid medication**											
*Blood pressure lowering medications*											
NZ/European	428 (35.2)	32	539 (35.2)	0	712 (50.8)	30	910 (63.5)	0	999 (69.5)	0	<0.0001
Māori	26 (43.3)	0	21 (25.6)	0	49 (48.5)	1	79 (57.2)	0	121 (57.1)	0	<0.0001
Pacific	16 (50.0)	0	30 (27.0)	0	94 (49.2)	6	168 (62.2)	0	250 (68.1)	0	<0.0001
Asian/other	6 (30.0)	0	14 (38.9)	0	73 (47.1)	7	163 (64.7)	0	305 (61.2)	0	<0.0001
Overall	476 (35.8)	32	604 (34.3)	0	942 (49.9)	52	1321 (63.0)	0	1675 (66.6)	0	<0.0001
*Antiplatelet agents*											
NZ/European	314 (27.4)	102	358 (23.4)	0	665 (48.2)	51	707 (49.3)	0	442 (30.7)	0	<0.0001
Māori	12 (21.4)	4	7 (8.5)	0	29 (29.6)	4	60 (43.5)	0	53 (25.0)	0	0.0197
Pacific	8 (28.6)	4	14 (12.6)	0	63 (33.5)	9	115 (42.6)	0	112 (30.5)	0	0.0204
Asian/other	4 (25.0)	4	8 (22.2)	0	56 (36.6)	9	117 (46.4)	0	152 (30.5)	0	0.423
Overall	338 (27.1)	114	387 (22.0)	0	832 (44.9)	83	999 (47.7)	0	759 (30.2)	0	<0.0001
*Anticoagulants*											
NZ/European	NA	0	33 (2.2)	0	137 (9.9)	50	117 (8.2)	0	224 (15.6)	0	<0.0001
Māori	NA	0	10 (12.2)	0	12 (12.2)	4	7 (5.1)	0	32 (15.1)	0	0.474
Pacific	NA	0	2 (1.8)	0	27 (14.1)	6	24 (8.9)	0	60 (16.3)	0	0.0008
Asian/other	NA	0	0 (0.0)	0	8 (5.2)	8	14 (5.6)	0	31 (6.2)	0	0.215
Overall	NA	0	45 (2.6)	0	185 (9.9)	78	162 (7.7)	0	347 (13.8)	0	<0.0001
*Lipid lowering drugs*											
NZ/European	NA	0	NA	0	213 (15.6)	63	567 (39.5)	0	585 (40.7)	0	<0.0001
Māori	NA	0	NA	0	13 (13.4)	5	58 (42.0)	0	93 (43.9)	0	<0.0001
Pacific	NA	0	NA	0	19 (10.4)	15	117 (43.3)	0	176 (48.0)	0	<0.0001
Asian/other	NA	0	NA	0	27 (18.0)	12	116 (46.0)	0	219 (44.0)	0	<0.0001
Overall	NA	0	NA	0	272 (14.4)	106	858 (40.9)	0	1073 (42.7)	0	<0.0001
**Management**											
*Admission to hospital within 28 days of stroke onset*											
NZ/European	768 (61.5)	0	1088 (71.0)	0	1283 (89.7)	0	1291 (90.0)	0	1423 (99.0)	0	<0.0001
Māori	46 (76.7)	0	73 (89.0)	0	99 (97.1)	0	124 (89.9)	0	208 (98.1)	0	<0.0001
Pacific	22 (68.8)	0	87 (78.4)	0	188 (95.4)	0	259 (95.9)	0	365 (99.5)	0	<0.0001
Asian/other	14 (70.0)	0	28 (77.8)	0	157 (96.9)	0	230 (91.3)	0	492 (98.8)	0	<0.0001
Overall	850 (62.5)	0	1276 (72.5)	0	1755 (90.9)	0	1906 (90.9)	0	2488 (98.9)	0	<0.0001
*Admission to acute stroke unit*											
NZ/European	NA	0	NA	0	140 (9.8)	0	709 (50.8)	40	1152 (80.2)	1	<0.0001
Māori	NA	0	NA	0	32 (31.4)	0	61 (46.2)	6	161 (75.9)	0	<0.0001
Pacific	NA	0	NA	0	39 (19.8)	0	140 (54.1)	11	289 (78.7)	0	<0.0001
Asian/other	NA	0	NA	0	24 (14.8)	0	131 (54.1)	10	385 (77.3)	0	<0.0001
Overall	NA	0	NA	0	238 (12.3)	0	1041 (51.3)	67	1987 (79)	1	<0.0001
*Neuroimaging, CT/MRI*											
NZ/European	134 (18.9)	538	429 (38.9)	429	1236 (86.4)	14	1389 (97.1)	4	1423 (99.0)	0	<0.0001
Māori	18 (50.0)	24	50 (67.6)	8	99 (97.1)	0	134 (97.8)	1	209 (98.6)	0	<0.0001
Pacific	6 (42.9)	18	50 (57.5)	24	180 (91.4)	0	261 (97.4)	2	364 (99.2)	0	<0.0001
Asian/other	4 (40.0)	10	12 (42.9)	8	153 (95.0)	1	244 (96.8)	0	494 (99.2)	0	<0.0001
Overall	162 (11.9)	590	541 (41.9)	469	1694 (87.6)	4	2030 (97.2)	7	2490 (99.0)	0	<0.0001
**Pathological type of stroke**											
Ischaemic stroke	NA	0	NA	0	1380 (71.2)	0	1694 (80.8)	0	1982 (78.8)	0	<0.0001
Primary intracerebral haemorrhage	NA	0	NA	0	236 (12.2)	0	275 (13.1)	0	401 (15.9)	0	0.0002
Subarachnoid haemorrhage	90 (6.6)	0	76 (4.3)	0	96 (5.0)	0	87 (4.2)	0	118 (4.7)	0	0.038
Undetermined	NA	0	NA	0	226 (11.7)	0	40 (1.9)	0	14 (0.6)	0	<0.0001
**Capture-recapture, missing (%)**	131/677 (19.3%)		185/1449 (12.8%)		144/1938 (7.4%)		30/2096 (1.4%)			116/2515 (4.6%)	<0.0001

a Trend p-values across all ARCOS studies were obtained using the Cochran-Armitage trend test for categorical variables, and ANOVA for continuous variables. For variables with more than 2 categories (ethnicity and source of notification), the p values were obtained using the chi-squared test. Missing data have been excluded. Stroke events by ethnicity may not be equal the total overall number due to missing ethnicity data: 1981–1982 study—3.3%; 1991–1992 study—2.2%; 2002–2002 study—5.5%; 2011–2012 study—2.5%; and 2021–2022 study—1.8%. For capture-recapture analysis, the numerator is the number of missing cases which was obtained from the log-linear model fitted for each study, and the denominator is the number of cases collected.

**Fig. 1:**
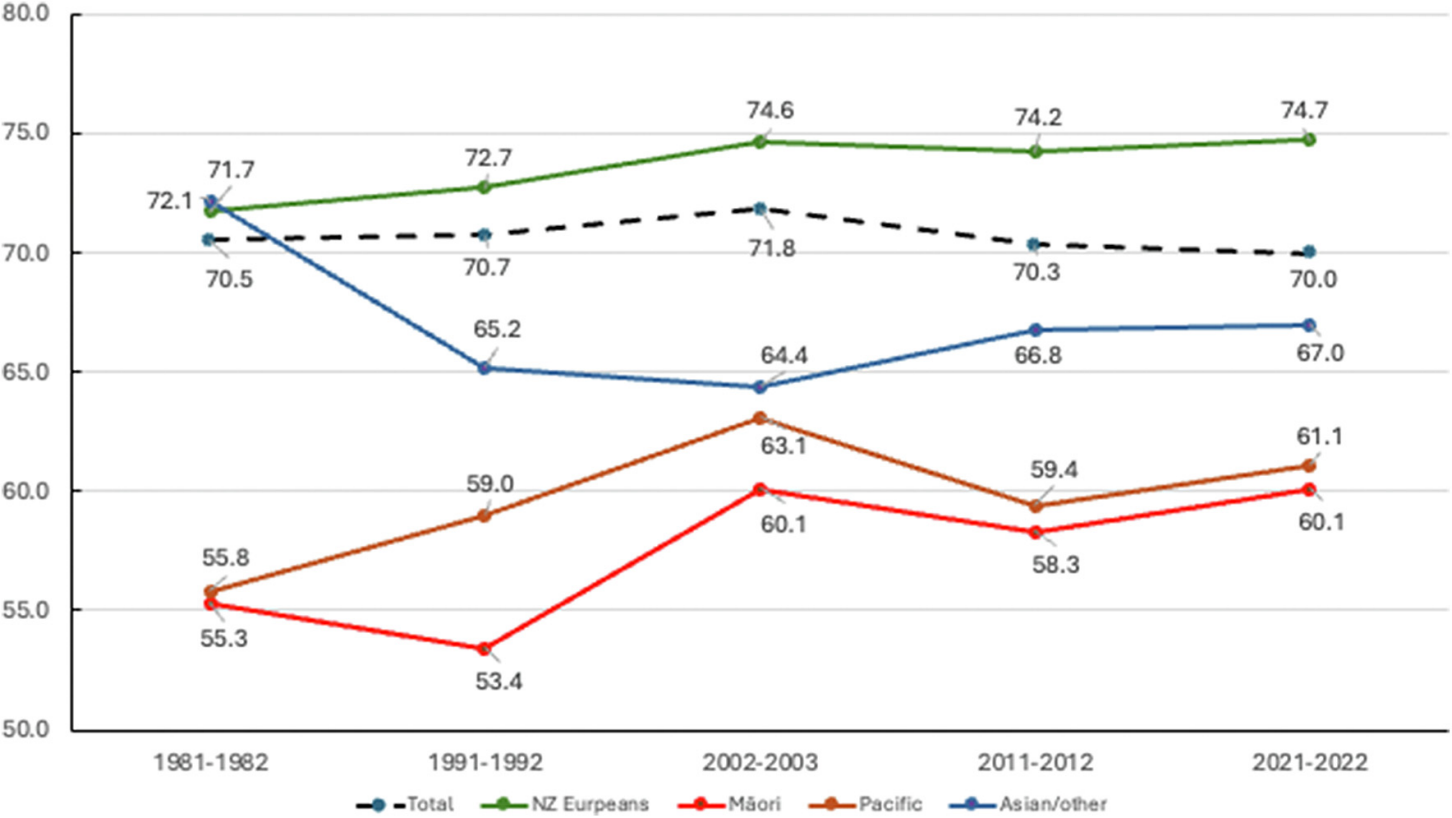
The overall mean age of individuals with incident stroke by ethnicity, 1981–2022∗. ∗Mean age of NZ Europeans was statistically significantly greater (p < 0.001) than the mean age of all other ethnic groups across all five ARCOS studies.

The proportion of strokes occurring in men increased from 48.7% in 1981–1982 to 52.2% in 2021–2022, and it was the lowest in 2002–2003 when it was 46.0%. The overall mean age of individuals with incident stroke has decreased from 1981–1982 (70.5 ± SD 14.2 years) to 2021–2022 (70.0 ± 15.5 years; p = 0.0092) mainly due to the reduction of the mean age of people within the Asian/other ethnic group (Fig. 1). In NZ Europeans, Māori, and Pacific people the mean age of individuals with incident stroke significantly increased from 1981–1982 (71.7 ± SD 13.7, 55.3 ± SD 14.3, and 55.8 ± SD 9.3, respectively) to 2021–2022 (74.7 ± SD 13.8, 60.1 ± SD 14.8, and 61.1 ± SD 15.0, respectively). Compared to NZ Europeans, the gap in the age of stroke onset in Māori (about 15 years younger), Pacific people (about 14 years younger), and Asian/other (about 8 years younger) did not significantly change over the study period. Similar mean age of differences was found in people with incident and recurrent strokes combined (Appendix Figure S1). The proportion of NZ Europeans among all individuals with first-ever stroke has almost linearly decreased from 1981–1982 (91.8%) to 2021–2022 (57.2%), with commensurate significant increases in the proportion of Māori (∼2-fold), Pacific (∼6-fold), and particularly Asian/other ethnic people (∼13-fold), which is likely to reflect the changing demographic of the Auckland population (Table 1).

Of 2515 stroke events in 2021–2022 (Table 1), the majority (78.8%) were IS, followed by PICH (15.9%), SAH (4.7%), and SUT (0.6%). From 1981–1982 to 2021–2022 there was a significant trend towards reduction of the proportion of SAH (6.6% vs 4.7%) and SUT (93.4% vs 0.5%), but from 2002–2003 to 2021–2022 there was an increase in the proportion of IS (71.2% vs 78.8%) and PICH (12.2% vs 15.9%) (Appendix Figure S2). Case ascertainment has significantly improved over the 40 years with the proportion of missing cases declining from 19.3% in the first ARCOS study (1981–1982) to an estimated 5.6% in the last ARCOS study (2021–2022).

### Stroke incidence

Although there was an overall 20.5% (95% CI 16.8; 23.5) decline in the age-standardised stroke incidence rates from 1981–1982 to 2021–2022 in NZ Europeans and Asian/other people, from 2011–2012 to 2021–2022 there was a significant increase in the age-standardised stroke incidence rate in Asian/other group (68/100,000; 95% CI 59; 79 and 117/100,000; 95% CI 106; 129, respectively), a not-statistically significant increase in Pacific people (197/100,000 [171; 226] and 202/100,000 [180; 226], respectively), and stagnation of the decline in Māori people (156/100,000 [128; 189] and 153/100,000 [131; 179], respectively) (Table 2, Appendix Figures S3 and S4A). Similar trends were observed for attack rates (Appendix Table S1 and Appendix Figure S4A). The absolute number of people affected by stroke has increased from 1981–1982 to 2021–2022 two-fold (Table 2, Appendix Figure S4B). In 2021–2022, the age-standardised incidence of stroke in Māori (153/100,000 [131; 179]) and Pacific (202/100,000 [180; 226]) was almost 1.5-fold greater than in NZ Europeans (111/100,000 [104; 118]) and almost 2.0-fold greater than in Asian/others (117/100,000 [95% CI 106; 129]). In 2021–2022, age-standardised stroke incidence rates in males (141/100,000 [95% CI 132; 150]) were greater than in females (109/100,000 [95% CI 102; 116]) overall in all age groups combined (Table 2, Fig. 1). These sex and ethnicity patterns were consistent over the last 30 years, but in 1981–1982 the highest age-standardised stroke incidence rates were observed in Asian/other albeit with a small sample size and small population denominator, followed by NZ Europeans, Pacific and Māori people. Analysis of age-standardised stroke attack rates is presented in Appendix p. 5.

**Table 2:** Crude, age-specific and age-standardised (to the age distribution of the WHO world population) annual stroke incidence rates (first-ever strokes) per 100,000 people-years in Auckland, New Zealand in each ARCOS study over the last 40 years (1981–1982, 1991–1992, 2002–2003, 2011–2012, and 2021–2022) by sex and ethnicity.

Age; sex and ethnicity	1981–1982			1991–1992			2002–2003			2011–2012			2021–2022			P for trend
	N	n	Rate (95% CI)	N	n	Rate (95% CI)	N	n	Rate (95% CI)	N	n	Rate (95% CI)	N	n	Rate (95% CI)	
**Males and females combined**																
15–64^a^	518,112	286	55 (46; 64)	624,828	347	56 (48; 63)	788,106	391	50 (45; 55)	956,037	528	55 (51; 60)	1,147,200	679	59 (55; 64)	
65–74	49,812	260	522 (432; 612)	56,388	373	661 (564; 759)	59,454	336	565 (505; 626)	95,190	363	381 (342; 421)	117,200	468	399 (363; 435)	
75–84	22,965	350	1524 (1298; 1750)	31,701	412	1300 (1139; 1460)	37,815	438	1158 (1050; 1267)	48,387	442	913 (828; 999)	59,800	530	886 (811; 962)	
85+	5691	134	2355 (1791; 2918)	8541	173	2026 (1684; 2367)	12,507	258	2063 (1811; 2315)	19,578	310	1583 (1407; 1760)	22,700	384	1692 (1522; 1861)	
Total	596,580	1030	173 (158; 188)	721,458	1305	181 (168; 194)	897,882	1423	158 (150; 167)	1,119,192	1643	147 (140; 154)	1,346,900	2061	153 (146; 160)	<0.0001
**Age-standardised**			**156 (143; 170)**			**156 (145; 167)**			**139 (132; 147)**			**119 (114; 125)**			**124 (119; 130)**	<0.0001
**Male**																
15–64^a^	256,500	164	64 (50; 78)	308,997	197	64 (52; 75)	380,139	216	57 (49; 64)	461,418	264	57 (50; 64)	570,700	394	69 (62; 76)	
65–74	22,251	158	710 (553; 867)	25,452	201	790 (628; 951)	28,173	198	703 (605; 801)	45,678	211	462 (400; 524)	56,700	277	489 (431; 546)	
75–84	8742	150	1716 (1328; 2104)	11,946	155	1298 (1038; 1557)	15,210	189	1243 (1065; 1420)	21,759	223	1025 (890; 1159)	27,400	244	891 (779; 1002)	
85+	1509	38	2518 (1386; 3651)	2421	34	1404 (932; 1876)	3633	64	1762 (1330; 2193)	6807	93	1366 (1089; 1644)	8400	157	1869 (1577; 2161)	
Total	289,002	510	176 (155; 198)	348,816	587	168 (150; 186)	427,155	667	156 (144; 168)	535,662	791	148 (137; 158)	663,200	1072	162 (152; 171)	0.0407
**Age-standardised**			**184 (163; 209)**			**167 (150; 185)**			**156 (144; 168)**			**129 (120; 138)**			**141 (132; 150)**	<0.0001
**Female**																
15–64^a^	261,612	122	47 (35; 58)	315,831	150	47 (38; 57)	407,967	175	43 (37; 49)	494,631	264	53 (47; 60)	576,400	285	49 (44; 55)	
65–74	27,561	102	370 (269; 472)	30,936	172	556 (437; 675)	31,281	138	441 (368; 515)	49,509	152	307 (258; 356)	60,500	191	316 (271; 360)	
75–84	14,223	200	1406 (1131; 1682)	19,755	257	1301 (1096; 1505)	22,605	249	1102 (965; 1238)	26,634	219	822 (713; 931)	32,300	286	885 (783; 988)	
85+	4182	96	2296 (1646; 2945)	6120	139	2271 (1833; 2709)	8874	194	2186 (1879; 2494)	12,771	217	1699 (1473; 1925)	14,300	227	1587 (1381; 1794)	
Total	307,578	520	169 (149; 190)	372,642	718	193 (175; 211)	470,727	756	161 (149; 172)	583,545	852	146 (136; 156)	683,500	989	145 (136; 154)	<0.0001
**Age-standardised**			**133 (118; 151)**			**143 (130; 158)**			**124 (115; 133)**			**110 (103; 119)**			**109 (102; 116)**	<0.0001
**European**																
15–64^a^	422,202	224	53 (43; 63)	459,267	233	51 (42; 59)	501,426	222	44 (38; 50)	450,759	252	56 (49; 63)	487,700	251	51 (45; 58)	
65–74	47,481	238	501 (411; 591)	52,125	341	654 (552; 756)	48,633	219	450 (391; 510)	64,806	239	369 (322; 416)	75,800	251	331 (290; 372)	
75–84	22,209	342	1540 (1309; 1771)	30,303	387	1277 (1114; 1441)	34,332	378	1101 (990; 1212)	35,916	354	986 (883; 1088)	43,000	362	842 (755; 929)	
85+	5577	130	2331 (1764; 2898)	8253	167	2024 (1675; 2372)	11,790	233	1976 (1722; 2230)	16,776	279	1663 (1468; 1858)	19,000	316	1663 (1480; 1847)	
Total	497,469	934	188 (171; 205)	549,948	1128	205 (189; 221)	596,181	1052	176 (166; 187)	568,257	1124	198 (186; 209)	625,500	1180	189 (178; 199)	0.728
**Age-standardised**			**153 (139–167)**			**150 (139; 163)**			**124 (116; 132)**			**122 (114; 130)**			**111 (104; 118)**	<0.0001
**Māori**																
15–64^a^	52,179	36	69 (37; 101)	63,762	48	75 (49; 101)	77,742	53	68 (50; 87)	88,470	74	84 (65; 103)	124,800	103	83 (67; 98)	
65–74	1266	6	474 (−62; 1010)	1344	3	223 (−29; 476)	2292	22	960 (559; 1361)	4452	22	494 (288; 701)	6900	39	565 (388; 743)	
75–84	336	4	1190 (−459; 2840)	429	8	1865 (573; 3157)	654	10	1529 (581; 2477)	1572	19	1209 (665; 1752)	2500	24	960 (576; 1344)	
85+	51	0	0	72	2	2778 (−1072; 6628)	144	4	2778 (56; 5500)	243	2	823 (−318; 1964)	500	5	1000 (123; 1877)	
Total	53,832	46	85 (51; 120)	65,607	61	93 (65; 121)	80,832	89	110 (87; 133)	94,737	117	123 (101; 146)	134,700	171	127 (108; 146)	0.0026
**Age-standardised**			**134 (78; 229)**			**168 (116; 241)**			**202 (157; 259)**			**156 (128; 189)**			**153 (131; 179)**	0.8597
**Pacific**																
15–64^a^	33,672	20	59 (23; 96)	64,506	51	79 (55; 103)	89,724	66	74 (56; 91)	107,688	126	117 (97; 137)	147,500	173	117 (100; 135)	
65–74	741	10	1350 (167; 2532)	2025	21	1037 (481; 1593)	3840	47	1224 (874; 1574)	6417	43	670 (470; 870)	9400	64	681 (514; 848)	
75–84	213	0	0	597	12	2010 (402; 3618)	1392	24	1724 (1034; 2414)	2679	29	1082 (689; 1476)	3900	43	1103 (773; 1432)	
85+	33	0	0	108	2	1852 (−715; 4418)	246	3	1220 (−160; 2600)	582	7	1203 (312; 2094)	1000	16	1600 (816; 2384)	
Total	34,659	30	87 (43; 130)	67,236	86	128 (96; 160)	95,202	140	147 (123; 171)	117,366	205	175 (151; 199)	161,800	296	183 (162; 204)	<0.0001
**Age-standardised**			**147 (80; 269)**			**225 (163; 310)**			**218 (183; 261)**			**197 (171; 226)**			**202 (180; 226)**	0.3139
**Asian & other combined**																
15–64^a^	10,059	6	60 (−8; 127)	37,293	15	40 (13; 68)	119,214	50	42 (30; 54)	309,123	76	25 (19; 30)	387,300	152	39 (33; 45)	
65–74	324	6	1852 (−244; 3947)	894	8	895 (−86; 1875)	4689	42	896 (625; 1167)	19,515	58	297 (221; 374)	25,100	114	454 (371; 538)	
75–84	207	4	1932 (−746; 4610)	372	5	1344 (166; 2522)	1437	21	1461 (836; 2086)	8220	40	487 (336; 637)	10,400	101	971 (782; 1161)	
85+	30	4	13,333 (−5146; 31,812)	108	2	1852 (−715; 4418)	327	7	2141 (555; 3727)	1971	22	1116 (650; 1583)	2200	47	2136 (1526; 2747)	
Total	10,620	20	188 (72; 305)	38,667	30	78 (40; 115)	125,667	120	95 (78; 113)	338,829	196	58 (50; 66)	425,000	414	97 (88; 107)	0.5226
**Age-standardised**			**360 (185; 701)**			**158 (92; 271)**			**166 (137; 202)**			**68 (59; 79)**			**117 (106; 129)**	<0.0001

N is the population at risk for a given age-group of the year of the study (denominator) and n is the number of incident strokes (nominator).

a 16–64 in 2011–2012 and 2021–2022.

**Fig. 2:**
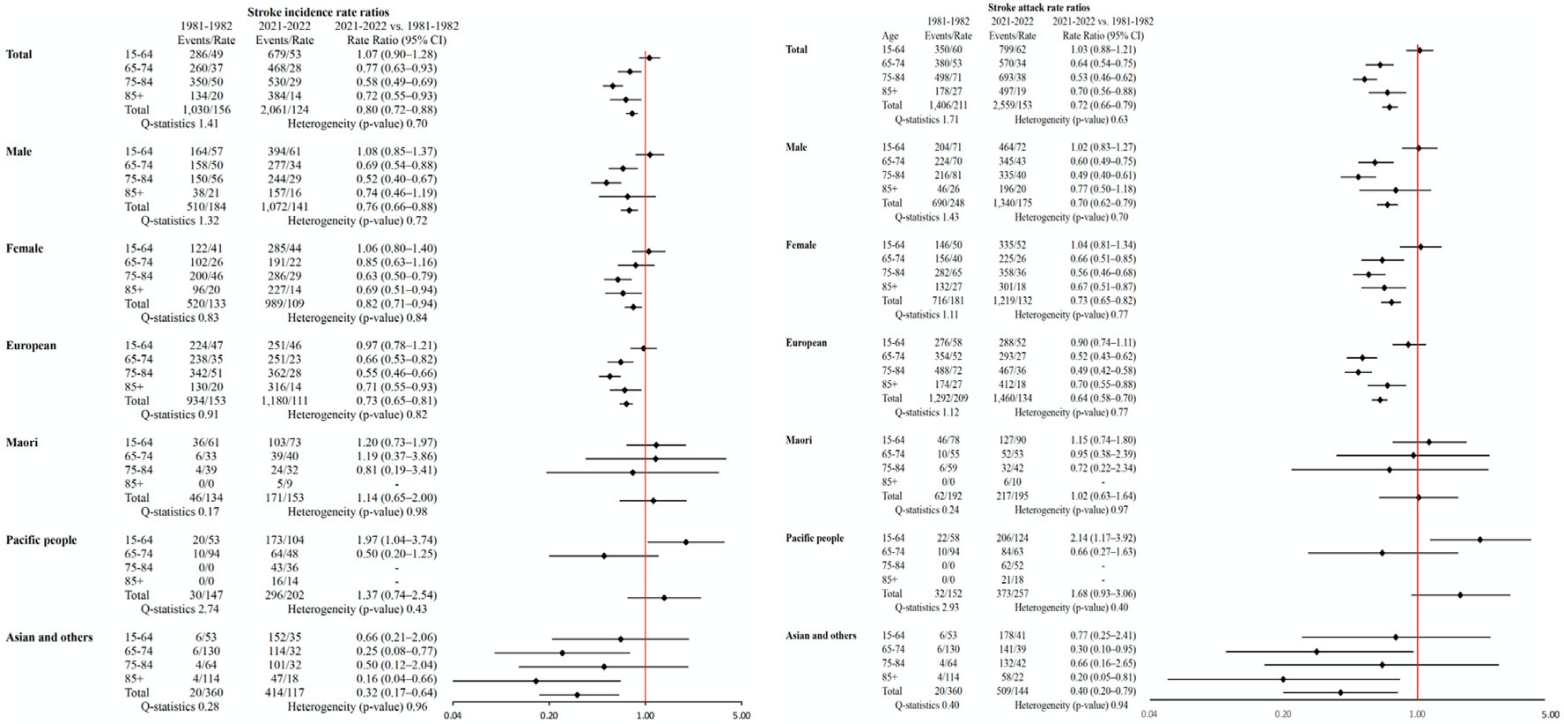
Forest plot of stroke incidence (first-ever) and attack rate ratios (RR) in 2021–2022 compared to 1981–1982 (reference) by ethnicity, with rates age-adjusted to the WHO world population∗. ∗Heterogeneity was measured by I^2^ test.

Age-standardised first-ever stroke incidence RRs between 1981–1982 and 2021–2022 (Fig. 2) decreased statistically significantly across all age groups, except for people aged 15–64 years (both males and females) where it was increased, albeit not statistically significantly, in both males (1.08 [0.85–1.37]) and females (1.06 [0.80–1.40]), with no significant heterogeneity across the age groups and studies, as suggested by the Q-statistics and I^2^ tests. Over the 40-year period, age-standardised stroke incidence RRs was statistically significantly increased in Pacific people aged 15–64 (RR 1.97 [1.04–3.74]), but statistically significantly reduced in European people aged 65–74, 75–84, and 85+ years (RRs 0.66 [0.53–0.82], 0.55 [0.46–0.66], and 0.71 [0.55–0.93], respectively). The average annual percent of stroke incidence rate from 2002–2003 to 2021–2022 increased by 1.48% (95% CI 0.53; 2.41) in people younger than 55 years but decreased by 1.57% (−1.95; −1.18) in people aged 55+ years (Appendix Figure S4A and B). There were diverging trends in RRs between NZ European, Asian/other people (reduction of RRs, though not statistically significant in Asian/other) and Māori and Pacific people (general increase of RRs, though not statistically significant). Similar to incident stroke, sex, ethnicity, and age group patterns of RRs were observed for stroke attack rates (Fig. 2).

### Stroke mortality, case-fatality and disability

In 2021–2022, the highest age-standardised 1-year stroke mortality rates (Table 3) were observed in Māori (52/100,000 [95% CI 39; 70] and Pacific people 53/100,000 [95% CI 42; 67]) followed by Asian/other (29/100,000 [95% CI 23; 35]) and NZ Europeans (22/100,000 [95% CI 19; 24]). Although a 3.5-fold reduction in the age-standardised all-cause one-year stroke mortality rates (Table 3) was observed over the last 40 years (from 98/100,000 [95% CI 88; 110] in 1981–1982 to 28/100,000 [95% CI 26; 31] in 2021–2022), with a similar pattern in males and females for each ethnic group, there was a noticeable slowing down in this reduction over the last decade (37/100,00 [95% CI 34; 40] in 2011–2012 and 28/100,000 [95% CI 26; 31] in 2021–2022). This was particularly noticeable in Māori (53/100,000 [95% CI 36; 77] in 2011–2012 and 52/100,000 [95% CI 39; 70] in 2021–2022) and Asian/other ethnic groups (27/100,000 [95% CI 21; 34] in 2011–2012 and 29/100,000 [95% CI 23; 35] in 2021–2022).

**Table 3:** Crude, age-specific and age-standardised (to the age distribution of the WHO world population) annual all-cause 1-year stroke mortality rates per 100,000 people in Auckland, New Zealand in each ARCOS study over the last 40 years (1981–1982, 1991–1992, 2002–2003, 2011–2012, and 2021–2022) by sex and ethnicity.

Age and ethnic group	1981–1982			1991–1992			2002–2003			2011–2012			2021–2022			Trend P value
	N	n	Rate (95% CI)	N	n	Rate (95% CI)	N	n	Rate (95% CI)	N	n	Rate (95% CI)	N	n	Rate (95% CI)	
**Total**																
15–64	518,112	104	20 (15; 26)	624,828	122	20 (16; 23)	788,106	89	11 (9; 14)	956,037	84	9 (7; 11)	1,147,200	85	7 (6; 9)	
65–74	49,812	168	337 (265; 409)	56,388	136	241 (196; 287)	59,454	99	167 (134; 199)	95,190	90	95 (75; 114)	117,200	85	73 (57; 88)	
75–84	22,965	266	1158 (961; 1355)	31,701	226	713 (615; 811)	37,815	232	614 (535; 692)	48,387	184	380 (325; 435)	59,800	166	278 (235; 320)	
85+	5691	126	2214 (1667; 2761)	8541	148	1733 (1422; 2044)	12,507	227	1815 (1579; 2051)	19,578	237	1211 (1056; 1365)	22,700	200	881 (759; 1003)	
Total	596,580	664	111 (99; 123)	721,458	632	88 (80; 95)	897,882	647	72 (67; 78)	1,119,192	595	53 (49; 57)	1,346,900	536	40 (36; 43)	<0.0001
**Age-standardised**			**98 (88; 110)**			**72 (67; 79)**			**57 (53; 62)**			**37 (34; 40)**			**28 (26; 31)**	<0.0001
**Male**																
15–64	256,500	58	23 (14; 31)	308,997	61	20 (15; 25)	380,139	48	13 (9; 16)	461,418	36	8 (5; 10)	570,700	43	8 (5; 10)	
65–74	22,251	98	440 (317; 564)	25,452	83	326 (242; 410)	28,173	59	209 (156; 263)	45,678	55	120 (89; 152)	56,700	48	85 (61; 109)	
75–84	8742	100	1144 (827; 1461)	11,946	102	854 (670; 1038)	15,210	88	579 (458; 699)	21,759	96	441 (353; 529)	27,400	76	277 (215; 340)	
85+	1509	28	1856 (884; 2828)	2421	22	909 (529; 1288)	3633	58	1596 (1186; 2007)	6807	73	1072 (826; 1318)	8400	77	917 (712; 1121)	
Total	289,002	284	98 (82; 114)	348,816	268	77 (67; 87)	427,155	253	59 (52; 67)	535,662	260	49 (43; 54)	663,200	244	37 (32; 41)	<0.0001
**Age-standardised**			**104 (88; 123)**			**76 (67; 87)**			**59 (52; 66)**			**39 (35; 44)**			**30 (26; 34)**	<0.0001
**Female**																
15–64	261,612	46	18 (10; 25)	315,831	61	19 (14; 25)	407,967	41	10 (7; 13)	494,631	48	10 (7; 12)	576,400	42	7 (5; 9)	
65–74	27,561	70	254 (170; 338)	30,936	53	171 (125; 217)	31,281	40	128 (88; 168)	49,509	35	71 (47; 94)	60,500	37	61 (41; 81)	
75–84	14,223	166	1187 (916; 1418)	19,755	124	628 (517; 738)	22,605	144	637 (533; 741)	26,634	88	330 (261; 399)	32,300	90	279 (221; 336)	
85+	4182	98	2343 (1687; 3000)	6120	126	2059 (1651; 2466)	8874	169	1904 (1617; 2192)	12,771	164	1284 (1088; 1481)	14,300	123	860 (708; 1012)	
Total	307,578	380	124 (106; 141)	372,642	364	98 (87; 108)	470,727	394	84 (75; 92)	583,545	335	57 (51; 64)	683,500	291	43 (38; 48)	<0.0001
**Age-standardised**			**92 (79; 106)**			**67 (60; 75)**			**55 (50; 61)**			**35 (32; 40)**			**27 (24; 31)**	<0.0001
**NZ European**																
15–64	422,202	76	18 (12; 24)	459,267	72	16 (12; 20)	501,426	44	9 (6; 11)	450,759	35	8 (5; 10)	487,700	15	3 (2; 5)	
65–74	47,481	156	329 (256; 401)	52,125	120	230 (183; 277)	48,633	60	123 (92; 155)	64,806	49	76 (54; 97)	75,800	43	57 (40; 74)	
75–84	22,209	258	1162 (961; 1362)	30,303	204	673 (578; 768)	34,332	193	562 (483; 641)	35,916	135	376 (312; 439)	43,000	101	235 (189; 281)	
85+	5577	122	2188 (1639; 2737)	8253	145	1757 (1437; 2076)	11,790	195	1654 (1422; 1886)	16,776	204	1216 (1049; 1383)	19,000	161	847 (716; 978)	
Total	497,469	612	123 (109; 137)	549,948	541	98 (89; 107)	596,181	492	83 (75; 90)	568,257	423	74 (67; 82)	625,500	320	51 (46; 57)	<0.0001
**Age-standardised**			**96 (86; 107)**			**67 (61; 74)**			**49 (45; 54)**			**35 (31; 39)**			**22 (19; 24)**	<0.0001
**Māori**																
15–64	52,179	14	27 (7; 47)	63,762	18	28 (15; 41)	77,742	16	21 (10; 31)	88,470	17	19 (10; 28)	124,800	19	15 (8; 22)	
65–74	1266	6	474 (−62; 1010)	1344	3	223 (−29; 476)	2282	7	305 (79; 532)	4452	4	90 (2; 178)	6900	15	217 (107; 327)	
75–84	336	4	1190 (−459; 2840)	429	9	2098 (727; 3469)	654	5	765 (94; 1435)	1572	9	573 (198; 947)	2500	14	560 (267; 853)	
85+	51	0	0	72	2	2778 (−1072; 6628)	144	2	1389 (−536; 3314)	243	3	1235 (−162; 2632)	500	3	600 (−79; 1279)	
Total	53,832	24	45 (19; 70)	65,607	32	49 (32; 66)	80,832	30	37 (24; 50)	94,737	33	35 (23; 47)	134,700	51	38 (27; 48)	0.2602
**Age-standardised**			**96 (47; 196)**			**133 (85; 209)**			**77 (50; 118)**			**53 (36; 77)**			**52 (39; 70)**	0.0046
**Pacific**																
15–64	33,672	12	36 (7; 64)	64,506	27	42 (26; 58)	89,724	17	19 (10; 28)	107,688	22	20 (12; 29)	147,500	35	24 (16; 32)	
65–74	741	4	540 (−208; 1288)	2025	10	494 (188; 800)	3840	21	547 (313; 781)	6417	20	312 (175; 448)	9400	15	160 (79; 240)	
75–84	213	0	0	597	10	1675 (135; 3215)	1392	19	1365 (751; 1979)	2679	16	597 (305; 890)	3900	16	410 (209; 611)	
85+	33	0	0	108	0	0	246	7	2846 (738; 4954)	582	10	1718 (653; 2783)	1000	9	900 (312; 1488)	
Total	33	16	46 (14; 78)	67,236	47	70 (48; 92)	95,202	64	67 (51; 84)	117,366	68	58 (44; 72)	161,800	75	46 (36; 57)	0.1124
**Age-standardised**			**69 (30; 160)**			**127 (81; 198)**			**124 (96; 162)**			**74 (58; 95)**			**53 (42; 67)**	<0.0001
**Asian/other**																
15–64	10,059	2	20 (−19; 59)	37,293	5	13 (2; 25)	119,214	11	9 (4; 15)	309,123	10	3 (1; 5)	387,300	16	4 (2; 6)	
65–74	324	2	617 (−593; 1827)	894	3	336 (−44; 715)	4689	8	171 (52; 289)	19,515	16	82 (42; 122)	25,100	12	48 (21; 75)	
75–84	207	4	1932 (−746; 4610)	372	3	806 (−106; 1719)	1437	10	696 (265; 1127)	8220	24	292 (175; 409)	10,400	35	337 (225; 448)	
85+	30	4	13,333 (−5146; 31,812)	108	1	926 (−889; 2741)	327	8	2446 (751; 4142)	1971	20	1015 (570; 1459)	2200	27	1227 (764; 1690)	
Total	10,620	12	113 (23; 203)	38,667	12	31 (13; 49)	125,667	37	29 (20; 39)	338,829	70	21 (16; 25)	425,000	90	21 (17; 26)	0.0002
**Age-standardised**			**238 (102; 557)**			**70 (37; 132)**			**64 (45; 91)**			**27 (21; 34)**			**29 (23; 35)**	<0.0001

N is the population at risk for a given age-group of the year of the study (denominator) and n is the number of fatal strokes by 1 years after stroke onset (nominator).

**Fig. 3:**
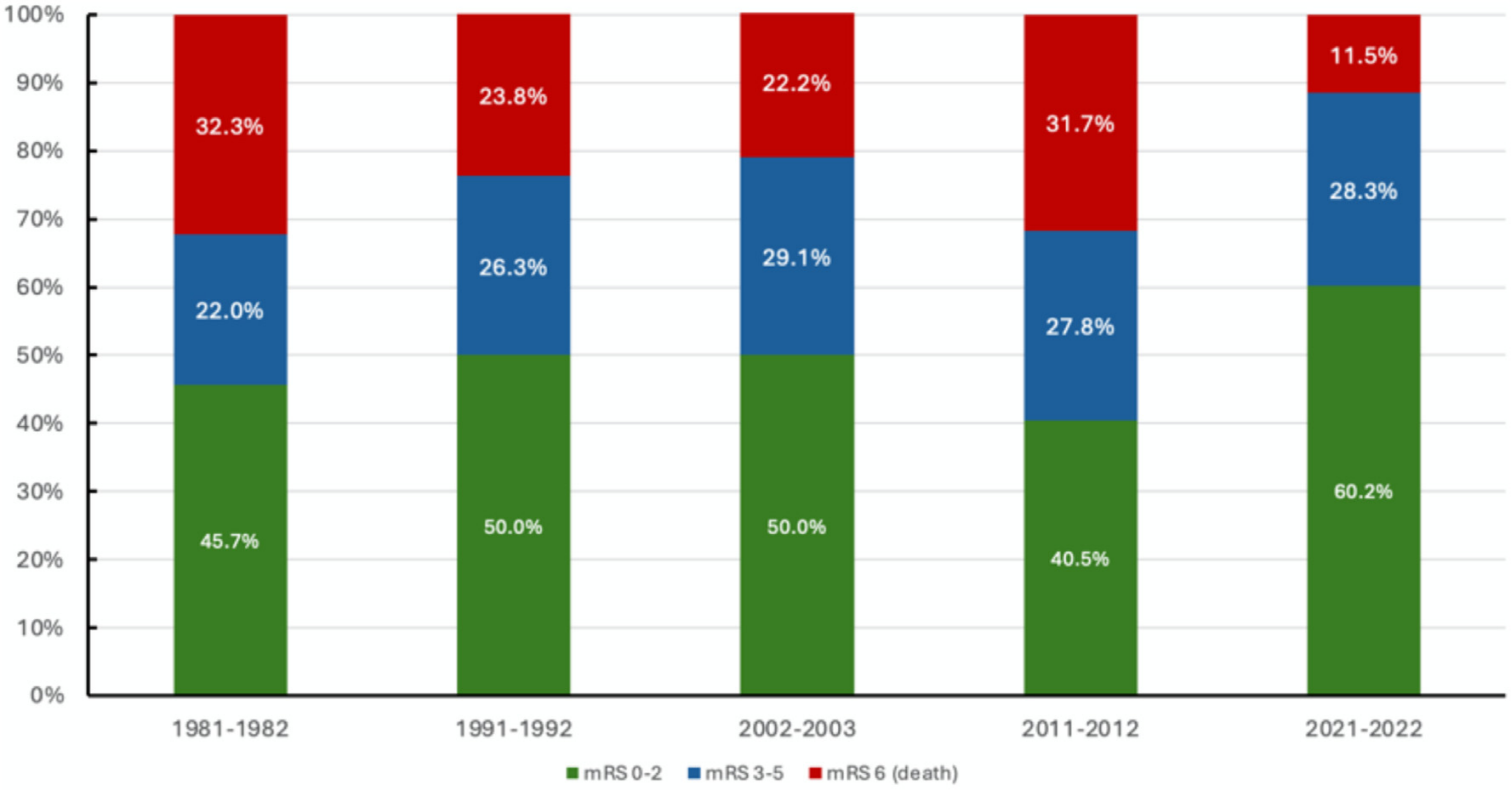
Trends in 28-day stroke case-fatality and disability (good and poor functional outcome)∗ in the Greater Auckland Region, 1981–2022. ∗Good functional outcome—modified Rankin Score 0–2. Poor functional outcome modified Rankin Score 3–5 (p for trend is <0.001 for mRS 0–2, 3–5, and 6).

Among individuals with first-ever stroke, 28-day stroke case-fatality reduced by almost three times (from 31.6% [95% CI 27.6; 35.7] in 1981–1982 to 11.4% [10.0; 12.7] in 2021–2022) in both males (from 26.4% [20.9; 31.8] to 9.3% [7.6; 11.1]) and females (from 36.8% [30.9; 42.7] to 13.5% [11.4; 15.7]) across all age groups (Table 4, Fig. 3, Appendix Table S2). Similar decreases were also observed in NZ Europeans (from 31.0% [26.8; 35.3] to 11.4% [9.6; 13.3]), Māori (from 30.4% [11.6; 49.3] to 14.0% [8.8; 19.2]), and Pacific people (from 40.0% [15.1; 64.9] to 10.5% [7.0; 14.0]); Table 4 whereas in the Asian/other ethnic group, the 28-day case fatality reduced from 50.0% (18.9; 81.1) to 10.6% (7.7; 13.6). In the 2021–2022 ARCOS study, Māori people aged 75–84 years old, and older females (85+ years old) had the highest 28-day case-fatality (Table 4, Appendix Table S2). Moreover, unlike incidence rates, the case-fatality rates dropped the most among younger age groups (15–64 and 65–74).

**Table 4:** 28-day fatal (case-fatality) and disability outcomes (good outcome [modified Rankin Score 0–2] and poor outcome [modified Rankin Score 3–5]), with 95% CI, of first-ever stroke in each ARCOS study over the last 40 years (1981–1982, 1991–1992, 2002–2003, 2011–2012, and 2021–2022) by age groups and ethnicity.

Age and ethnic group	1981–1982			1991–1992			2002–2003			2011–2012			2021–2022			Trend P value
	N	n	% (95% CI)	N	n	% (95% CI)	N	n	% (95% CI)	N	n	% (95% CI)	N	n	% (95% CI)	
**Case-fatality**																
*By age groups*																
15–64^a^	282	72	25.5 (18.3; 32.7)	340	72	21.2 (16.5; 25.8)	334	56	16.8 (12.8; 20.8)	280	57	20.4 (15.6; 25.1)	677	48	7.1 (5.2; 9)	<0.0001
65–74	258	78	30.2 (22.3; 38.2)	366	61	16.7 (12.2; 21.2)	279	47	16.8 (12.5; 21.2)	198	46	23.2 (17.3; 29.1)	467	35	7.5 (5.1; 9.9)	<0.0001
75–84	344	122	35.5 (28.3; 42.6)	400	97	24.3 (19.6; 28.9)	381	71	18.6 (14.7; 22.5)	259	87	33.6 (27.8; 39.3)	529	69	13 (10.2; 15.9)	<0.0001
85+	132	56	42.4 (30.5; 54.4)	165	72	43.6 (35.3; 52)	228	97	42.5 (36.1; 49)	188	103	54.8 (47.7; 61.9)	382	85	22.3 (18.1; 26.4)	<0.0001
Total	1016	328	32.3 (28.2; 36.4)	1271	302	23.8 (21.1; 26.4)	1222	271	22.2 (19.8; 24.5)	925	293	31.7 (28.7; 34.7)	2055	237	11.5 (10.2; 12.9)	<0.0001
*By ethnicity*																
NZ/European	922	292	31.7 (27.4; 35.9)	1103	253	22.9 (20.2; 25.7)	924	205	22.2 (19.5; 24.9)	700	212	30.3 (26.9; 33.7)	1176	137	11.6 (9.8; 13.5)	<0.0001
Māori	46	14	30.4 (11.6; 49.2)	58	15	25.9 (13.8; 37.9)	73	18	24.7 (14.8; 34.5)	56	18	32.1 (19.9; 44.4)	170	24	14.1 (8.9; 19.4)	0.0087
Pacific	30	12	40 (15.2; 64.8)	81	29	35.8 (24.5; 47.1)	114	24	21.1 (13.6; 28.5)	85	27	31.8 (21.9; 41.7)	295	31	10.5 (7; 14)	<0.0001
Asian/other	18	10	55.6 (23.1; 88)	29	5	17.2 (2.4; 32.1)	101	16	15.8 (8.7; 23)	83	35	42.2 (31.5; 52.8)	414	45	10.9 (7.9; 13.9)	<0.0001
**Good functional outcome**^a^																
*By age groups*																
15–64^a^	282	148	52.5 (44.2; 60.7)	340	199	58.5 (52.2; 64.8)	334	197	59 (53.7; 64.3)	280	152	54.3 (48.4; 60.1)	677	503	74.3 (71; 77.6)	<0.0001
65–74	258	124	48.1 (39.4; 56.7)	366	213	58.2 (51.2; 65.2)	279	162	58.1 (52.3; 63.9)	198	97	49 (42; 56)	467	315	67.5 (63.2; 71.7)	<0.0001
75–84	344	142	41.3 (33.9; 48.6)	400	183	45.8 (39.3; 52.2)	381	183	48 (43; 53)	259	92	35.5 (29.7; 41.4)	529	275	52 (47.7; 56.2)	0.0281
85+	132	50	37.9 (26.2; 49.6)	165	40	24.2 (15.7; 32.8)	228	53	23.2 (17.8; 28.7)	188	34	18.1 (12.6; 23.6)	382	144	37.7 (32.8; 42.6)	0.1751
Total	1016	464	45.7 (41.3; 50)	1271	635	50 (46.4; 53.6)	1222	595	48.7 (45.9; 51.5)	925	375	40.5 (37.4; 43.7)	2055	1237	60.2 (58.1; 62.3)	<0.0001
*By ethnicity*																
NZ/European	922	430	46.6 (42.1; 51.2)	1103	568	51.5 (47.6; 55.4)	924	469	50.8 (47.5; 54)	700	297	42.4 (38.8; 46.1)	1176	723	61.5 (58.7; 64.3)	<0.0001
Māori	46	20	43.5 (23.2; 63.7)	58	21	36.2 (21.1; 51.3)	73	31	42.5 (31.1; 53.8)	56	21	37.5 (24.8; 50.2)	170	101	59.4 (52; 66.8)	0.0024
Pacific	30	10	33.3 (9.5; 57.2)	81	31	38.3 (25.4; 51.1)	114	51	44.7 (35.6; 53.9)	85	32	37.6 (27.3; 48)	295	162	54.9 (49.2; 60.6)	0.0008
Asian/other	18	4	22.2 (0; 49.4)	29	15	51.7 (27.5; 75.9)	101	42	41.6 (32; 51.2)	83	25	30.1 (20.2; 40)	414	251	60.6 (55.9; 65.3)	<0.0001
**Poor functional outcome**^a^																
*By age groups*																
15–64^a^	282	62	22 (15.1; 28.8)	340	69	20.3 (15.2; 25.4)	334	81	24.3 (19.7; 28.8)	280	71	25.4 (20.3; 30.5)	677	126	18.6 (15.7; 21.5)	0.3251
65–74	258	56	21.7 (14.6; 28.8)	366	92	25.1 (19.4; 30.8)	279	70	25.1 (20; 30.2)	198	55	27.8 (21.5; 34)	467	117	25.1 (21.1; 29)	0.3670
75–84	344	80	23.3 (16.9; 29.6)	400	120	30 (24.7; 35.3)	381	127	33.3 (28.6; 38.1)	259	80	30.9 (25.3; 36.5)	529	185	35 (30.9; 39)	0.0008
85+	132	26	19.7 (10.1; 29.3)	165	53	32.1 (24.1; 40.1)	228	78	34.2 (28.1; 40.4)	188	51	27.1 (20.8; 33.5)	382	153	40.1 (35.1; 45)	0.0003
Total	1016	224	22 (18.4; 25.7)	1271	334	26.3 (23.4; 29.2)	1222	356	29.1 (26.6; 31.7)	925	257	27.8 (24.9; 30.7)	2055	581	28.3 (26.3; 30.2)	0.0013
*By ethnicity*																
NZ/European	922	200	21.7 (17.9; 25.5)	1103	282	25.6 (22.5; 28.7)	924	250	27.1 (24.2; 29.9)	700	191	27.3 (24; 30.6)	1176	316	26.9 (24.3; 29.4)	0.0097
Māori	46	12	26.1 (8.1; 44)	58	22	37.9 (22.8; 53)	73	24	32.9 (22.1; 43.7)	56	17	30.4 (18.3; 42.4)	170	45	26.5 (19.8; 33.1)	0.3518
Pacific	30	8	26.7 (4.3; 49.1)	81	21	25.9 (15.9; 36)	114	39	34.2 (25.5; 42.9)	85	26	30.6 (20.8; 40.4)	295	102	34.6 (29.1; 40)	0.1754
Asian/other	18	4	22.2 (0; 49.4)	29	9	31 (11.3; 50.8)	101	43	42.6 (32.9; 52.2)	83	23	27.7 (18.1; 37.3)	414	118	28.5 (24.2; 32.9)	0.1879

a Missing good/poor functional outcome data (n, %) at discharge/28-days post-stroke onset: 1981–1982—14 (1.4%), 1991–1992—34 (2.65), 2002–2003—201 (14.1%), 2011–2012—718 (43.7%), 2021–2022—6 (0.3%).

We also observed a significant improvement in discharge/28-day good functional outcomes in first-ever stroke survivors (Table 4, Fig. 3). Good functional outcome (mRS 0–2) statistically significantly increased from 45.7% (95% CI 41.3; 50.0) in 1981–1982 to 60.2% (58.1; 62.3) in 2021–2022 (p for trend <0.0001), especially noticeable in people younger than 75 years of age, and to a lesser extent in people aged 75–84 years (p = 0.0281), with no significant changes in people 85 years or older (p = 0.1751). As shown in Table 4, good functional outcome improved over the last 40 years across all ethnic groups, especially in Pacific people (33.3% [9.5; 57.2] in 1981–1982 to 54.9% [49.2; 60.6]) and Asian/other (22.2% [0.0; 49.4] and 60.6% [55.9; 65.3], respectively). The proportion of stroke survivors with poor functional outcome was significantly increased over the study period only in 75–84 and 85+ years old people (from 23.3% [16.9; 29.6] and 19.7% [10.1; 29.3] in 1981–1982 to 35.0% [30.9; 39.0] and 40.1% [35.1; 45.0] in 2021–2022, respectively [p for trend <0.001]) and only in NZ Europeans (from 21.7% [17.9; 25.5] in 1981–1982 to 26.9% [24.3; 29.4] in 2012–2022; p for trend <0.01).

### Pooled analysis with previous population-based studies

In comparison with a recent systematic review of the stroke incidence trends among younger and older populations,^3^ our updated literature search did not yield additional prospective population-based studies that would have met our inclusion criteria. Therefore, our pooled analysis included six previous population-based cohort studies^33^, ^34^, ^35^, ^36^, ^37^, ^38^ in trends in incidence of first-ever stroke during the early 21st century and separately for the younger (aged <45^38^ or <55^33^, ^34^, ^35^, ^36^, ^37^ years) and older (aged ≥45^38^ or ≥55^33^, ^34^, ^35^, ^36^, ^37^ years) individuals. All six studies also included trends of 28-day or 1-month case-fatality (for consistency of the reporting, 1-month case-fatality was equalised to and reported as 28-). Since only one of the studies^33^ had trends for functional outcome, our pooled analysis was limited to changes in incidence and 28-day case-fatality. Based on the pooled estimates (Appendix Figure S5A), stroke incidence rates among younger individuals aged <55 years tended to increase on average by 1.6% (95% CI −0.3, 3.5) per year during the early 21st century although this did not reach statistical significance. In contrast, the incidence rates among older adults reduced on average by 2.2% (1.3, 3.1) over the same period (Appendix Figure S5B). Even though we observed substantial heterogeneity between the findings of included studies (I^2^ = 77.9% for the young age group and I^2^ = 91.0% for the older age group), none of the studies showed significantly decreasing trends for the younger population or significantly increasing trends for the older population (Appendix Figure S4C). In addition, our pooled analysis showed an average of 1.7% [0.8, 2.5] annual decrease in 28-day stroke case-fatality rates among all ages during the early 21st century with modest between-cohort heterogeneity (I^2^ = 35.6%).

## Discussion

This is the first prospective mixed-method population-based study of long-term (40-year) trends of stroke incidence, 1-year mortality, 28-day case fatality, and discharge/28-day disability in a large (>1.3 million adults) ethnically mixed study population.^18^^,^^39^ While consistent with the encouraging significant reduction in stroke incidence and mortality rates across the first four ARCOS studies (1981–1982, 1991–1992, 2002–2003, and 2011–2012) reported previously,^16^ the current ARCOS V study (2021–2022) demonstrated a further deepening of ethnic disparities, with a trend, though not statistically significant, towards higher age-standardised stroke incidence rates over the last decade. From 1981–1982 to 2021–2022 we observed a statistically significant decrease in the age-standardised stroke incidence rates in NZ Europeans and Asian/other people, and some increase, albeit not statistically significant, in the age-standardised incidence rates in Māori and Pacific. Over the last four decades, the mean age of individuals at incident stroke onset has statistically significantly reduced from 70.5 years (SD 14.2) in 1981–1982 to 70.0 (15.5) years in 2021–2022 despite the noticeable ageing of the New Zealand population.^40^ This trend was more pronounced over the last two decades and in Asian/other. Moreover, the stable gap between mean age at stroke onset of Māori, Pacific and Asian/other ethnic group people and NZ Europeans (approximately 15, 14, and 8 years younger, respectively) persisted. The drop in stroke incidence in Asian/other people (mostly Chinese/East Asians) in 2011–2012 is likely to be related to the immigration of younger, presumably generally healthy, Asian people to NZ over the last two decades.^41^ The significantly improved disability level and reduced 28-day case-fatality over the last four decades was particularly noticeable since 2010 when revascularisation therapy was widely introduced in hospitals of the Greater Auckland Region, NZ.

Our findings on the trends towards increasing stroke incidence rates in younger adults (statistically significant increase in people younger than 55 years and not statistically significant increase in people aged <64 years) and decreasing rates in older adults (statistically significant decrease in people 55+ years old and 65+ years) are consistent with the results of the pooled analyses of methodologically comparable population-based stroke incidence and outcome studies conducted in other HICs over the last three decades and suggest that currently used primary stroke/CVD prevention strategies are not sufficiently effective.^3^ Indeed, a large proportion of young adults with arterial hypertension^42^ and dyslipidaemia^43^ remain undertreated owing at least partly to the widespread use of absolute CVD risk^44^ treatment thresholds.^3^ The widespread use of high CVD risk treatment thresholds in NZ over the last 20 years has likely led many people with hypertension^42^ and hyperlipidaemia^43^ being deprived of appropriate pharmacological treatment because they did not reach the recommended CVD risk threshold for treatment,^3^ as per the current high CVD risk prevention guidelines.^45^ Although high risk thresholds in CVD screening were supported by the WHO in 2007,^46^ a more recent (2021) WHO Health Evidence Network Synthesis Report 71 - that included their own meta-analysis of randomised clinical trials - concluded that screening for CVD risk and CVD risk factors has had no impact on lowering CVD morbidity and mortality in the general population, and even increased mortality.^47^ Based on their analysis, the WHO Health Evidence Network Synthesis group experts recommended that Member States of the WHO European Region review existing systematic population-level screening programmes for CVD risk and risk factors, avoid initiating new such programmes, and consider alternative methods to achieve the desired outcomes in reducing the CVD burden.^47^

Our findings demonstrate that, similar to most other HICs in the world,^1^ in absolute terms, the burden of stroke in the Greater Auckland Region, NZ continues to grow significantly, largely due to population growth and ageing of the population, although improved awareness of stroke might have contributed to better ascertainment of minor strokes. By extrapolating our findings to the whole of NZ in 2023 (4,203,000 residents aged ≥15 years old)^48^ and conservatively assuming that the mean survival time after stroke in NZ in 2002–2003 (9.0 years in males and 8.2 years in females)^49^ remains unchanged up to 2023, we estimate that there are currently about 6400 first-ever strokes in NZ annually. Overall, combining first-ever and recurrent events, we estimate 8000 new stroke events per year (12,000 including TIA; unpublished data). Among these 8000 events, approximately 900 people die from stroke within the first 28 days and 1700 people die within a year after stroke. These estimates are close to those estimated for NZ in the GBD 2021 Study.^50^

The increase in the proportion of PICH in 2021–2022 and the corresponding reduction in the proportion of stroke of undetermined pathological type compared to previous study periods are likely a reflection of the greater use of brain neuroimaging within the first days of stroke admission to the hospital (99.0% in 2021–2022) and a greater proportion of Asian/other ethnic groups known for the high predisposition to PICH in the study region.^51^^,^^52^ However, the significant reduction in the proportion of SAH from 6.6% in 1981–1982 to 4.7% in 2021–2022 may also be related to the reduction in smoking in NZ, especially over the last two decades.^53^ While the proportion of strokes in NZ Europeans almost linearly declined from 1981–1982 to 2021–2022, the increase in the proportion of strokes in Māori, Pacific and Asian/other people over the last 40 years further contributes to the increased gap in stroke burden between NZ Europeans and other ethnic groups in NZ. In addition, the ongoing high and increasing prevalence of pre-existing hypertension (especially in Pacific people), myocardial infarction (especially in NZ Europeans) and type 2 diabetes mellitus (especially in Pacific and Asian/other people) in people with stroke also indicate ongoing ethnic disparities. Inadequate control of these pre-morbid conditions in these populations indicates the need for targeted prevention strategies. Despite the relatively high prevalence of pre-morbid health conditions and risk factors in people with stroke, their level of pre-stroke management remains suboptimal, with only 66.6% of individuals with hypertension receiving blood pressure lowering medications, 42.7% receiving lipid lowering medications, 30.2% of individuals with previous atherosclerotic CVD (including ischaemic stroke) receiving antiplatelet medications and only 13.8% of individuals with pre-stroke AF receiving anticoagulant medications. Our findings indicate that pre-morbid stroke prevention pharmacological treatment is particularly poor in Pacific people.

There has been a large increase in the proportion of people treated in acute stroke units (79.0% in 2021–2022), having early neuroimaging (99.0% in 2021–2022), along with a significant reduction in early (28-days) stroke case-fatality over 40 years across all ethnic groups. These changes were especially noticeable over the last two decades after the wider introduction of acute stroke units, wider use of oral anticoagulants and revascularisation therapy.^54^, ^55^, ^56^ These positive changes in the organisation of acute stroke care were also likely influenced by a shift of early post-stroke functional outcomes towards milder strokes: the proportion of people discharged from hospitals with no disabilities (mRS 0–2) increased from 45.7% to 60.2% over the last 40 years. Similarly, 28-day stroke case-fatality among individuals with first-ever stroke showed almost linear reductions across all age/sex groups and ethnicity. A trend towards lowered post-stroke disability and improved stroke survival has also been observed in other research.^57^, ^58^, ^59^, ^60^, ^61^, ^62^ Although positive, improved survival puts additional pressure on rehabilitation services, especially community rehabilitation. Even now in high-income countries, community rehabilitation is available for only a limited period of time (usually 2–3 months) and for only about one-third of post-discharge stroke survivors with disabilities,^63^^,^^64^ owing to their limited number, financial and personal constraints.^65^ Availability and access to community rehabilitation services in low- and middle-income countries are even more limited.^66^ Under these circumstances, home-based^67^ and self-management rehabilitation^68^^,^^69^ with freely available 24/7 audio-visual tutorial materials that show promising results in improving post-stroke recovery^70^^,^^71^ offer a tangible solution. However, a radical reduction in stroke burden requires a complex, multisectoral (government and non-government organisations) approach covering all aspects of stroke prevention, care and rehabilitation, as outlined in the recent World Stroke Organization—Lancet Neurology Commission on stroke.^72^

Our study has a number of limitations. As reported in our previous ARCOS I–IV publication,^16^ in the 1981–1982 and 2002–2003 studies, only one ethnicity was collected for participants in each study, preventing us from applying prioritised ethnicity classification in these two studies. We believe the effect of the possible misclassification bias was not large as most of the ethnic priority classifications in the 1991–1992 and 2011–2012 studies were also based on the first ethnicity self-identified by the study participants, with less than 10% indicating more than one ethnicity. Although we applied the same criteria for cardiovascular risk factors recorded across all studies, we acknowledge that the accuracy of information about these risk factors was prone to some degree of variation and, therefore, should be interpreted with caution. There was a difference in case ascertainment between the 1981–1982 and 1991–1992 studies (sampling of 50% and 25% of GP records) and 2002–2003, 2011–2012, 2021–2022 studies (complete case ascertainment). Although this difference may have resulted in more complete/accurate case ascertainment in the last three studies, we believe it did not significantly influence our results because capture-recapture analysis showed almost complete case ascertainment across all five studies. Furthermore, even though the first three studies were one of the largest population-based epidemiological studies of stroke in the world at the time of their conduct, disaggregating the estimates into age and ethnicity subgroups did result in a small number of events, thus raising a possibility of type 1 error. Therefore, the ethnic and age-specific estimates for the first three studies (particularly the first two studies) should be interpreted with caution. There was also a significant proportion of missing data on functional outcomes at discharge/28-days post-stroke in the 2011–2012 study. We also were not able to collect data on ungrouped mRS scores, thus preventing overall mRS score estimates. However, excluding the 2011–2012 data on functional outcomes did not significantly change the trend analysis. In addition, the consistency of the assessment procedures, diagnostic criteria, the implementation of quality control procedures during the five studies, together with the decreased estimated number of missing stroke cases as determined by capture-recapture analyses, reassure us that case-ascertainment was high across all these studies. Due to the demographic particulars of the NZ population, generalisability of the study findings are limited to the HICs with predominantly Caucasian/White population. Finally, due to the lack of reliable verification of pathological types of stroke in the 1981–1982 and 1991–1992 studies, analysis of changes in pathological types of stroke was limited to the 2002–2003 and 2011–2012 ARCOS studies.

In conclusion, we showed an overall lowering in age-standardised stroke incidence rates and significant improvements in 28-day stroke case-fatality and disability level in the Greater Auckland Region over the last 40 years. However, over the last decade (2011–2022) we also observed a statistically significant increase in age-standardised stroke incidence rates in Asian/other people and people younger than 55 years old as well as worsening ethnic disparities in stroke burden in NZ. In terms of the number of people who have developed a new stroke or died from a stroke, the burden of stroke in the study population has substantially risen. Urgent measures to improve primary stroke prevention are required.

## Contributors

VLF designed the study, obtained funding (study PI), participated in the supervision of the study, planned analyses of the data, and wrote the first draft of the manuscript. IR did the pooled analysis of current and previous study estimates and helped create the tables and graphs. RK (study Co-PI) participated in the study design and together with BN supervised the day-to-day running of the study and contributed to discussions. RK, BA, PAB, SB-C, JD, DE, VP, AR, AS, E-ST, BTA, BT participated in the design of the study and gaining funding. PAB, YR, VLF assessed the diagnosis of stroke events. VP was responsible for performing analyses and contributed to discussions. IZ participated in the data analysis. All authors contributed to the critical revision of the manuscript for important intellectual content.

## Data sharing statement

The authors confirm that the data supporting the findings of this study are available within the article and its Supplementary Materials.

## Declaration of interests

VLF declares that he is a CEO of the New Zealand Stroke Education (charitable) Trust that provides online free of charge stroke self-management rehabilitation videos. CSA received research grants, consulting fees and honoraria from AstraZeneca, works as President-elect of World Stroke Organisation and Editor-in-Chief of Cerebrovascular Diseases; PAB is President Australia and New Zealand Association of Neurologists; AR received consulting fees from Australian Health Department, works in Stroke Foundation NZ, Australian and NZ Stroke Organisation, and World Stroke Organisation. Other authors declare no conflict of interest.
