# Supplementary material for: Trends in stroke incidence, death, and disability outcomes in a multi-ethnic population: Auckland regional community stroke studies (1981–2022)
Source: Lancet Reg Health West Pac. 2025 Mar 10;56:101508. doi: 10.1016/j.lanwpc.2025.101508 (PMC11938151; doi:10.1016/j.lanwpc.2025.101508)
Supplement: Appendix [file mmc1.pdf]

The Lancet Regional Health
Western Pacific

Appendix

STUDY METHODOLOGY 2

LITERATURE SEARCH AND POOLED ANALYSES 3

DEMOGRAPHIC CHARACTERISTICS OF THE STUDY POPULATION 4

AGE-STANDARDISED STROKE ATTACK RATES 5

APPENDIX TABLE 1. CRUDE, AGE-SPECIFIC AND AGE-STANDARDISED (TO THE AGE DISTRIBUTION OF THE WHO WORLD POPULATION) ANNUAL ATTACK (FIRST-EVER AND RECURRENT STROKES COMBINED) RATES PER 100,000 PEOPLE-YEARS IN AUCKLAND, NEW ZEALAND IN EACH ARCOS STUDY OVER THE LAST 40 YEARS (1981-1982, 1991-1992, 2002-2003, 2011-2012 AND 2021-2022) BY AGE, SEX AND ETHNICITY 6

APPENDIX TABLE 2. 28-DAY CASE FATALITY (%) AMONG FIRST-EVER (INCIDENT) STROKE CASES IN AUCKLAND, NEW ZEALAND IN EACH ARCOS STUDY OVER THE LAST 40 YEARS (1981-1982, 1991-1992, 2002-2003 AND 2011-2012) BY AGE, SEX AND ETHNICITY 8

APPENDIX TABLE 3. 28-DAY CASE FATALITY (%) AMONG FIRST-EVER AND RECURRENT STROKE CASES COMBINED IN AUCKLAND, NEW ZEALAND IN EACH ARCOS STUDY OVER THE LAST 40 YEARS (1981-1982, 1991-1992, 2002-2003 AND 2011-2012) BY AGE, SEX AND ETHNICITY 10

APPENDIX FIGURE 1. THE OVERALL MEAN AGE OF INDIVIDUALS WITH STROKE (FIRST-EVER AND RECURRENT STROKES COMBINED) BY ETHNICITY, 1981-2022 12

APPENDIX FIGURE 2. TRENDS IN THE PROPORTIONS (%) OF THE PATHOLOGICAL TYPES OF STROKE, 2002-2022 13

APPENDIX FIGURE 3. AGE-STANDARDISED STROKE INCIDENCE RATES PER 100,000/PERSON-YEARS (WITH 95% CI IN UPPER GRAPH) BY ETHNICITY, 1981-2022 14

APPENDIX FIGURE 4. TRENDS IN (A) AGE-STANDARDISED STROKE INCIDENCE AND ATTACK RATES AND (B) ABSOLUTE NUMBER OF INCIDENT AND TOTAL STROKES (INCIDENT AND RECURRENT COMBINED), 1981-2022 15

APPENDIX FIGURE 5A-C. FOREST PLOTS FOR POOLED ANALYSIS OF AVERAGE ANNUAL PERCENT CHANGES (AAPC) WITH 95% CIS OF A) FIRST-EVER STROKE INCIDENCE IN YOUNG PEOPLE, B) FIRST-EVER STROKE INCIDENCE IN OLDER PEOPLE, AND C) 1-MONTH CASE-FATALITY OF FIRST-EVER STROKES IN ALL AGES DURING THE EARLY 21ST CENTURY 16

Study methodology

To allow valid comparisons across the studies, acute stroke was defined using WHO clinical diagnostic criteria.[1] For the last ARCOS study (2021-2022) we also used (in a separate analysis) the new International Classification of Diseases 11th Revision (ICD-11)[2] tissue-based definitions of stroke in which TIAs with CT/MRI evidence of ischaemic brain lesions are classified as ischaemic strokes (IS). Across all five ARCOS studies, we registered first-ever and recurrent new strokes (any new stroke event 28 days after the index event) during the study period.

For the 1981-1982 and 1991-1992 ARCOS studies,[3] all hospitalised cases with new stroke in the Greater Auckland Region were identified through daily searches of all hospital admission lists of all public hospitals in the region. All death certificates and autopsy reports were checked monthly, and verbal autopsy methods was applied[4] to any event where stroke was mentioned as a cause of death. For identification of all new non-hospitalised and non-fatal stroke events during the study period, a cluster sample of 50% (1981-1982 study) and 25% (1991-1992 study) of all registered general physicians (GPs) was used to identify a representative sample of stroke events in the study population. GPs were asked to refer patients suspected of having a stroke but who were cared for either at home or in a rest home or private hospital. The total numbers of cases were calculated based on the appropriate sampling fraction. Stroke events in patients enrolled with these selected practitioners were included in the study regardless of their source of referral.[5] All deceased cases were identified through hospital admissions and discharge reports, systematic searches of post-mortem reports and death certificates in national registries.[6] Data for fatal non-hospitalised SAH cases were collected from medical records only.

In the two most recent studies (2002-2003 and 2011-2012)[7,8] no cluster sampling was used and instead the whole study population was monitored for new stroke events. To ensure a complete prospective case-ascertainment in 2002-2003 and 2011-2012 studies we undertook daily searches of hospital presentation data, where a diagnosis suggesting stroke or transient ischaemic attack (TIA) was recorded for all public hospitals and emergency departments, CT/MRI records and hospital discharge registers; weekly checks of all private hospitals, rest homes, and community health services (general practices, hospital outpatient clinics and rehabilitation centres); quarterly checks of coroner/autopsy records, death certificates (from the Registrar of Births, Deaths and Marriages) to identify people who had died with any mention of stroke, and NZ Health Information Service data of all fatal and non-fatal stroke/TIA cases in the study population.

In the first two ARCOS studies (1981-1982 and 1991-1992), CT/MRI neuroimaging was not widely available (done in 11.9% and 41.9% stroke patients, respectively) therefore of the three pathological types of stroke (ischaemic stroke [IS], intracerebral haemorrhage [ICH] and subarachnoid haemorrhage [SAH]) we were able to reliably identify only cases of SAH defined as “an abrupt onset of a severe headache and/or impaired consciousness or focal neurological signs associated with at least one of the following findings: uniform blood staining of the cerebrospinal fluid; CT evidence of blood in the subarachnoid space; cerebral angiographic identification of an aneurysm or arteriovenous malformation, or identification of SAH at surgery or at autopsy. This definition excludes PICH with extension into the subarachnoid space and subarachnoid bleeding due to trauma, neoplasms, or infections”.[9] For the first two studies we also did not register aetiological subtypes of IS. For the last three ARCOS studies (2002-2003, 2011-2012 and 2021-2022), the rate of CT/MRI neuroimaging within the first seven days of stroke onset was high (87.6%, 97.2% and 99.0%, respectively), therefore we analysed trends by pathological type of stroke (IS, ICH, SAH) and five major aetiological subtype of IS

using TOAST criteria (large artery atherosclerosis [LAA], cardioembolism [CE], small artery occlusion [SAO], IS of other determined cause [SOC], and IS of undetermined cause [SUC]) over the last two decades only.[10]

For fatal strokes we searched National Birth, Death and Marriage Records and identified death certificates with stroke mentioned as the first or secondary cause of death in the residents of the Greater Auckland Region over the study period. To ascertain the cause of death in all these cases we used the WHO verbal autopsy methodology.[4] Regardless of the ICD version used for coding stroke events (ICD-9 or ICD-10), all ICD codes relevant to stroke were ascertained using the same WHO diagnostic criteria for stroke and stroke pathological types. To allow valid comparisons, all new stroke cases, including suspected strokes and TIAs, were ascertained by stroke physicians of the study Stroke Adjudication Committee. Similar to the methodology used in ARCOS III and ARCOS IV studies, stroke risk factors prevalence, management variations and medication use in the ARCOS V study were ascertained via hospital and outpatient medical records. Baseline variables obtained from medical records included socio-demographics, history of the stroke, medical and neurological history, family history, risk factors, results of routine cardiovascular and neurological examinations, pre-morbid disability, level of dependency at discharge and a functional and self-care assessment. Disability level at 28 days after stroke onset (as measured by modified Rankin Scale [mRS] in individuals with first-ever stroke only)[11] was based on in-hospital assessment, discharge summary or by a telephone assessment if the patient had been discharged home or into residential care.

Similar to our previous report,[6] completeness of case ascertainment based on the sources of notification was determined using capture-recapture techniques.[12] This involved conducting log-linear modelling assuming a Poisson distribution (unadjusted for sample procedures),[13] and used the four main sources of notification: hospital, general practitioner, death certificate and other sources. The final model with the least deviance for all studies included the main effects of the four sources and the 3-way interaction between hospital, GP and death certificate.

Literature search and pooled analyses

Based on a recent systematic review and meta-analysis[14] of trends in stroke incidence differences between young and older individuals during the early 21st century, we extracted other 'ideal' prospective, population-based studies reporting first-ever stroke incidence trends by age in defined geographical regions of HICs. To verify the previous search strategy and identify studies published after the review,[14] we followed the Preferred Reporting Items for Systematic Reviews and Meta-analysis (PRISMA) reporting guidelines[15] and searched for additional studies in Medline, Scopus, Google Scholar, and PubMed with the terms "stroke", "cerebrovascular events/disease", "registry", "survey", "epidemiology", "incidence", case-fatality", "mortality", "morbidity", "ethnic/racial", "disability", "trend(s)", and "population or community based". Our structured search included studies published between 1 January 2000 and 1 June 2024 without language restrictions. Similar to the previous systematic review,[14] the inclusion criteria were: (1) population-based study design (including both hospitalised and non-hospitalised cases) in a high-income setting; (2) data on all first-ever stroke types (ischaemic stroke, primary intracerebral haemorrhage, subarachnoid haemorrhage and undetermined pathological type); (3) availability of incidence rates or number of stroke cases and persons at risk for younger (aged <45 or <55 years) and older age groups (aged ≥45 or ≥55 years) in

two data points of the same population. Since we primarily focused on the time trends during the 21st century, we only included the studies with the first data collection between 1990 and 2010 and the last data collection after 2010. Two authors (IR and VLF) also searched reference lists of all relevant publications, and any disagreements in selected studies and extracted data were resolved by discussion. If multiple data points fulfilled the inclusion criteria, we used the consecutive period closest to 2000 as the beginning and the last available consecutive period as the end of incidence trend estimation. Moreover, we gathered the data on trends in 28-day case-fatality and disability rates among those with first-ever stroke.

Demographic characteristics of the study population

The study population (Greater Auckland Region) was relatively stable in terms of migration since 1980th: the net migration rate was from -2.0 to -1.1 per 1000 people in 1981-82, 3.7-5.2/1000 in 1991-1992, 5.8-6.7/1000 in 2002-2003, 3.6-3.8/1000 in 2011-2012, and 2.6-2.5 in 2021-2022.[16] Overall, there were 7,462 new first-ever-in-a-lifetime stroke events registered across the five studies (Table 1). From 1981-1982 to 2021-2022, there was a significant 19.8% increase in hospitalisation of people with acute stroke, reaching almost complete hospitalisation (99.8%) in the last study. Over the 40-year study period among individuals with a new stroke, we observed a significant 5.7% increase in the prevalence of hypertension (≥140/90 on admission, previously diagnosed hypertension or blood pressure lowering drug use before admission), mainly due to the increase in the prevalence of hypertension in NZ Europeans and Asian/other ethnicities, with no significant change in the prevalence of hypertension in Pacific and significant reduction in Māori people. There was a significant 6.6% increase in the prevalence of pre-morbid myocardial infarction across all ethnic groups by NZ Europeans, a significant 16.5% increase in the prevalence of pre-morbid type 2 diabetes mellitus (mainly due to the increase in the number of people of NZ Europeans, Pacific and Asian/other ethnicity), but a significant 6.2% decrease in the prevalence of pre-morbid previous stroke, though only in NZ Europeans. Data on the prevalence of atrial fibrillation (AF), both persistent and paroxysmal, pre-morbid and diagnosed first at admission, were not available for the first two ARCOS studies (1981-1982 and 1991-1992). Over the last 20 years, the prevalence of AF in individuals with stroke was significantly reduced by about 2%, but only because of the corresponding reduction in Asian/other ethnic group people; in stroke individuals of other ethnicity changes in the prevalence of AF were not statistically significant, and slightly prevailed in NZ Europeans, Māori and Pacific people over that in Asian/other ethnic groups. The prevalence of pre-morbid current smoking in individuals with stroke has increased statistically significantly by (from 27.7% in 1981-1982 to 32.6% in 2021-2022; P for trend <0.0001), especially in NZ Europeans (26.7% and 34.1%; p<0.0001), while it has reduced in Pacific (37.5% and 33.2%; p<0.0001) and Asian/other people (25.0 and 21.5%; p<0.0001) and remained very high in Māori (53.3% and 46.9%; p=0.241).

The male to female ratio did not significantly change over the study period, with males constituting overall 62.9% of all new first-ever stroke events. The overall mean age of individuals with stroke has significantly decreased from 1981-1982 (71.2±13.3 years) to 2021-2022 (70.7±15.2 years; p<0.0001), but we observed diverging trends in the mean age of individuals with stroke by ethnicity: in NZ Europeans, Māori and Pacific people the mean age has significantly increased over the 40-year study period by 3.2, 4.7, and 6.3 years, respectively, while in Asian/other ethnic groups over the same time period it significantly decreased by 4.2 years. The gap in the age of stroke onset between NZ Europeans and Māori (about 15 years younger in the former), Pacific people (about 14 years younger than in NZ

Europeans) and Asian/other (about 8 years younger than in NZ Europeans) did not significantly change over the study period. While the proportion of NZ Europeans among all individuals with new stroke has almost linearly decreased from 1981-1982 (91.8%) to 2021-2022 (57.2%), the proportion of Māori, Pacific and particularly Asian/other ethnic group people has significantly increased.

Over the last 40 years we observed a statistically significant increase in the pre-stroke use of blood pressure lowering medications across all ethnicities, antiplatelet agents (mainly in NZ Europeans, Māori and Pacific people), and anticoagulants (mainly in NZ Europeans and Pacific people). The pre-stroke use of lipid lowering medications, hospital admission within the first 7 days of stroke onset, brain CT/MRI within the first 7 days of stroke admission, and treatment in acute stroke units also increased statistically significantly across all ethnic groups. All ethnic group individuals with acute stroke experienced statistically significant trends in the reduction of case-fatality within the first 28 days after stroke onset, especially noticeable over the last decade. Similarly, there was a statistically significant trend in the reduction of time from stroke onset to the assessment of the study participants by research staff. Of 2,061 new first-ever stroke events in 2021-2022, 78.8% constituted ischaemic stroke (IS), 15.9% - primary intracerebral haemorrhage (PICH), 4.7% - subarachnoid haemorrhage (SAH), and 0.6% - stroke of undetermined pathological type (SUT). There was a significant trend towards reduction of the proportion of SAH and SUT, but an increase in the proportion of IS and PICH. Case-ascertainment has significantly improved over the 40-year period, and in the last ARCOS study (2021-2022) we estimated that only 116 stroke cases might have been missed.

Age-standardised stroke attack rates

The age-standardised stroke attack rates (Appendix Table 1, appendix Figure 4) were similar to the age-standardised incidence rate patterns in trends from 1981-1982 to 2021-2022, with an overall stagnation in the age-standardised stroke attack rates over the last decade (153/100,000 [95% CI 147; 160] in 2011-2012 and 153/100,00 [95% CI 147; 159] in 2021-2022). Between 2011-2012 and 2021-2022, there was also a non-significant trend towards an increase in the age-standardised attack rates in males (167/100,000 [95% CI 157; 178] and 175/100,000 [95% CI 166; 185]) and Māori (192/100,000 [95% CI 161; 229] and 195/100,000 [95% CI 170;225], respectively). Moreover, the age-standardised attack rates increased by 60.0% in the Asian/other ethnic group over the last decade from 90/100,000 (95% CI 79; 101) in 2011-2012 to 144/100,000 (95% CI 132; 158). This increase was particularly evident in people aged 15-64, 65-74, and 75-85 years.

Appendix Table 1. Crude, age-specific and age-standardised (to the age distribution of the WHO world population) annual attack (first-ever and recurrent strokes combined) rates per 100,000 people-years in Auckland, New Zealand in each ARCOS study over the last 40 years (1981-1982, 1991-1992, 2002-2003, 2011-2012 and 2021-2022) by age, sex and ethnicity

Age; sex and ethnicity group	1981-1982			1991-1992			2002-2003			2011-2012			2021-2022			*P* value for trend
	N	n	Rate (95% CI)	N	n	Rate (95% CI)	N	n	Rate (95% CI)	N	n	Rate (95% CI)	N	n	Rate (95% CI)	
**Total**																
15-64*	518112	350	68 (58;78)	624828	433	69 (61; 77)	788106	484	61 (56; 67)	956037	626	65 (60; 71)	1147200	799	70 (65; 74)	
65-74	49812	380	763 (654; 871)	56388	512	908 (795; 1021)	59454	460	774 (703; 844)	95190	476	500 (455; 545)	117200	570	486 (446; 526)	
75-84	22965	498	2169 (1899; 2438)	31701	611	1927 (1730; 2125)	37815	667	1764 (1630; 1898)	48387	598	1236 (1137; 1335)	59800	693	1159 (1073; 1245)	
85+	5691	178	3128 (2478; 3778)	8541	247	2892 (2460; 3324)	12507	390	3118 (2809; 3428)	19578	448	2288 (2076; 2500)	22700	497	2189 (1997; 2382)	
Total	596580	1406	236 (218; 253)	721458	1803	250 (235; 265)	897882	2001	223 (213; 233)	1119192	2148	192 (184; 200)	1346900	2559	190 (183; 197)	<0.0001
**Age-standardised**			**211 (196; 228)**			**213 (201; 227)**			**193 (185; 202)**			**153 (147; 160)**			**153 (147; 159)**	<0.0001
**Male**																
15-64*	256500	204	80 (64; 95)	308997	252	82 (69; 94)	380139	273	72 (63; 80)	461418	323	70 (62; 78)	570700	464	81 (74; 89)	
65-74	22251	224	1007 (820; 1193)	25452	290	1139 (947; 1332)	28173	273	969 (854; 1084)	45678	272	595 (525; 666)	56700	345	608 (544; 673)	
75-84	8742	216	2471 (2005; 2937)	11946	252	2109 (1768; 2451)	15210	277	1821 (1607; 2036)	21759	305	1402 (1244; 1559)	27400	335	1223 (1092; 1354)	
85+	1509	46	3048 (1803; 4294)	2421	41	1694 (1175; 2212)	3633	95	2615 (2089; 3141)	6807	138	2027 (1689; 2366)	8400	196	2333 (2007; 2660)	
Total	289002	690	239 (214; 264)	348816	835	239 (218; 261)	427155	918	215 (201; 229)	535662	1038	194 (182; 206)	663200	1340	202 (191; 213)	<0.0001
**Age-standardised**			**248 (223; 276)**			**236 (216; 258)**			**214 (200; 228)**			**167 (157; 178)**			**175 (166; 185)**	<0.0001
**Female**																
15-64*	261612	146	56 (43; 69)	315831	181	57 (47; 67)	407967	211	52 (45; 59)	494631	303	61 (54; 68)	576400	335	58 (52; 64)	
65-74	27561	156	566 (440; 692)	30936	222	718 (587; 848)	31281	187	598 (512; 683)	49509	204	412 (356; 469)	60500	225	372 (323; 420)	
75-84	14223	282	1983 (1655; 2310)	19755	359	1817 (1577; 2058)	22605	390	1725 (1554; 1897)	26634	293	1100 (974; 1226)	32300	358	1108 (994; 1223)	
85+	4182	132	3156 (2395; 3918)	6120	206	3366 (2799; 3934)	8874	295	3324 (2945; 3704)	12771	310	2427 (2157; 2698)	14300	301	2105 (1867; 2343)	
Total	307578	716	233 (209; 257)	372642	968	260 (239; 281)	470727	1083	230 (216; 244)	583545	1110	190 (179; 201)	683500	1219	178 (168; 188)	<0.0001
**Age-standardised**			**181 (163; 201)**			**190 (175; 206)**			**173 (162; 184)**			**140 (132; 149)**			**132 (125; 140)**	<0.0001
**NZ European**																
15-64*	422202	276	65 (54; 76)	459267	293	64 (55; 73)	501426	269	54 (47; 60)	450759	288	64 (57; 71)	487700	288	59 (52; 66)	
65-74	47481	354	746 (636; 855)	52125	459	881 (764; 997)	48633	303	623 (553; 693)	64806	312	481 (428; 535)	75800	293	387 (342; 431)	
75-84	22209	488	2197 (1922; 2473)	30303	579	1911 (1709; 2113)	34332	568	1654 (1518; 1790)	35916	470	1309 (1190; 1427)	43000	467	1086 (988; 1185)	
85+	5577	174	3120 (2464; 3776)	8253	241	2920 (2476; 3364)	11790	345	2926 (2617; 3235)	16776	400	2384 (2151; 2618)	19000	412	2168 (1959; 2378)	
Total	497469	1292	260 (240; 280)	549948	1572	286 (267; 305)	596181	1485	249 (236; 262)	568257	1470	259 (245; 272)	625500	1460	233 (221; 245)	<0.0001
**Age-standardised**			**209 (193; 226)**			**206 (193; 220)**			**171 (162; 180)**			**154 (145; 163)**			**134 (126; 142)**	<0.0001
**Māori**																
15-64*	52179	46	88 (52; 124)	63762	58	91 (63; 119)	77742	60	77 (58; 97)	88470	88	99 (79; 120)	124800	127	102 (84; 119)	
65-74	1266	10	790 (98; 1482)	1344	8	595 (183; 1008)	2282	24	1047 (628; 1466)	4452	25	562 (341; 782)	6900	52	754 (549; 958)	
75-84	336	6	1786 (-235; 3806)	429	14	3263 (1554; 4973)	654	15	2294 (1133; 3454)	1572	24	1527 (916; 2138)	2500	32	1280 (837; 1723)	
85+	51	0	0	72	2	2778 (-1072; 6628)	144	5	3472 (429; 6516)	243	4	1646 (33; 3259)	500	6	1200 (240; 2160)	
Total	53832	62	115 (75; 156)	65607	104	129 (104; 153)	80832	104	129 (104; 153)	94737	141	149 (124; 173)	134700	217	161 (140; 183)	0.0032
**Age-standardised**			**192 (122; 304)**			**254 (188; 341)**			**247 (196; 311)**			**192 (161; 229)**			**195 (170; 225)**	0.9754

**Pacific**																
15-64*	33672	22	65 (27; 104)	64506	65	101 (74; 127)	89724	88	98 (78; 119)	107688	155	144 (121; 167)	147500	206	140 (121; 159)	
65-74	741	10	1350 (167; 2532)	2025	33	1630 (899; 2360)	3840	67	1745 (1327; 2163)	6417	64	997 (753; 1242)	9400	84	894 (703; 1085)	
75-84	213	0	0	597	13	2178 (536; 3819)	1392	39	2802 (1922; 3661)	2679	45	1680 (1189; 2171)	3900	62	1590 (1194; 1985)	
85+	33	0	0	108	2	1852 (-715; 4418)	246	8	3252 (998; 5506)	582	15	2577 (1273; 3882)	1000	21	2100 (1202; 2998)	
Total	34659	32	92 (47; 138)	67236	113	168 (131; 205)	95202	202	212 (183; 241)	117366	279	238 (210; 266)	161800	373	231 (207; 254)	<0.0001
**Age-standardised**			**152 (85; 274)**			**291 (220; 384)**			**329 (284; 382)**			**275 (244; 310)**			**257 (232; 285)**	0.0111
**Asian/other**																
15-64*	10059	6	60 (-8; 127)	37293	17	46 (17; 74)	119214	64	54 (41; 67)	309123	95	31 (25; 37)	387300	178	46 (39; 53)	
65-74	324	6	1852 (-244; 3947)	894	12	1342 (268; 2416)	4689	55	1173 (863; 1483)	19515	74	379 (293; 466)	25100	141	562 (469; 654)	
75-84	207	4	1932 (-746; 4610)	372	5	1344 (166; 2522)	1437	34	2366 (406; 3161)	8220	58	706 (524; 887)	10400	132	1269 (1053; 1486)	
85+	30	4	13333 (-5146; 31812)	108	2	1852 (-715; 4418)	327	10	3058 (1163; 4954)	1971	29	1471 (936; 2007)	2200	58	2636 (1958; 3315)	
Total	10620	20	188 (72; 305)	38667	36	93 (54; 132)	125667	163	130 (110; 150)	338829	256	76 (66; 85)	425000	509	120 (109; 130)	0.6019
**Age-standardised**			**360 (185; 701)**			**194 (122; 310)**			**234 (197; 277)**			**90 (79; 101)**			**144 (132; 158)**	<0.0001

Appendix Table 2. 28-day case fatality (%) among first-ever (incident) stroke cases in Auckland, New Zealand in each ARCOS study over the last 40 years (1981-1982, 1991-1992, 2002-2003 and 2011-2012) by age, sex and ethnicity

Age and	1981-1982			1991-1992			2002-2003			2011-2012			2021-2022			Trend
ethnic group	N	n	% (95% CI)	N	n	% (95% CI)	N	n	% (95% CI)	N	n	% (95% CI)	N	n	% (95% CI)	*P* value
**Total**																
15-64*	286	72	25.2% (18% - 32.3%)	346	72	20.8% (16.2% - 25.4%)	391	56	14.3% (10.8% - 17.8%)	528	57	10.8% (8.1% - 13.4%)	679	48	7.1% (5.1% - 9%)	<.0001
65-74	258	76	29.5% (21.6% - 37.4%)	371	61	16.4% (12% - 20.9%)	336	47	14% (10.3% - 17.7%)	363	46	12.7% (9.2% - 16.1%)	468	35	7.5% (5.1% - 9.9%)	<.0001
75-84	346	120	34.7% (27.6% - 41.8%)	413	97	23.5% (19% - 28%)	438	71	16.2% (12.8% - 19.7%)	442	87	19.7% (16% - 23.4%)	530	69	13% (10.2% - 15.9%)	<.0001
85+	134	56	41.8% (29.9% - 53.6%)	175	72	41.1% (33.2% - 49.1%)	258	97	37.6% (31.7% - 43.5%)	310	103	33.2% (28% - 38.5%)	384	85	22.1% (18% - 26.3%)	<.0001
Total	1024	324	31.6% (27.6% - 35.7%)	1305	302	23.1% (20.6% - 25.7%)	1423	271	19% (17% - 21.1%)	1643	293	17.8% (16% - 19.7%)	2061	237	11.5% (10.1% - 12.9%)	<.0001
**Male**																
15-64*	164	36	22% (13% - 30.9%)	196	36	18.4% (12.6% - 24.2%)	216	29	13.4% (8.9% - 18%)	264	26	9.8% (6.3% - 13.4%)	394	23	5.8% (3.5% - 8.2%)	<.0001
65-74	158	44	27.8% (17.9% - 37.8%)	202	36	17.8% (11.3% - 24.3%)	198	26	13.1% (8.4% - 17.8%)	211	28	13.3% (8.7% - 17.9%)	277	16	5.8% (3% - 8.5%)	<.0001
75-84	148	42	28.4% (18.1% - 38.7%)	155	38	24.5% (17.1% - 32%)	189	28	14.8% (9.7% - 19.9%)	223	46	20.6% (15.3% - 25.9%)	244	31	12.7% (8.5% - 16.9%)	<.0001
85+	38	12	31.6% (10.6% - 52.6%)	34	15	44.1% (27.4% - 60.8%)	64	26	40.6% (28.6% - 52.7%)	93	29	31.2% (21.8% - 40.6%)	157	32	20.4% (14.1% - 26.7%)	0.0045
Total	508	134	26.4% (20.9% - 31.8%)	587	125	21.3% (17.5% - 25%)	667	109	16.3% (13.5% - 19.2%)	791	129	16.3% (13.7% - 18.9%)	1072	102	9.5% (7.8% - 11.3%)	<.0001
**Female**																
15-64*	122	36	29.5% (18% - 41%)	150	36	24% (16.7% - 31.3%)	175	27	15.4% (10.1% - 20.8%)	264	31	11.7% (7.9% - 15.6%)	285	25	8.8% (5.5% - 12.1%)	<.0001
65-74	100	32	32% (19% - 45%)	169	25	14.8% (9% - 20.6%)	138	21	15.2% (9.2% - 21.2%)	152	18	11.8% (6.7% - 17%)	191	19	9.9% (5.7% - 14.2%)	<.0001
75-84	198	78	39.4% (29.7% - 49.1%)	258	59	22.9% (17.3% - 28.5%)	249	43	17.3% (12.6% - 22%)	219	41	18.7% (13.5% - 23.9%)	286	38	13.3% (9.3% - 17.2%)	<.0001
85+	96	44	45.8% (31.7% - 60%)	141	57	40.4% (31.4% - 49.4%)	194	71	36.6% (29.8% - 43.4%)	217	74	34.1% (27.8% - 40.4%)	227	53	23.3% (17.8% - 28.9%)	<.0001
Total	516	190	36.8% (30.9% - 42.7%)	718	177	24.7% (21.2% - 28.1%)	756	162	21.4% (18.5% - 24.4%)	852	164	19.2% (16.6% - 21.9%)	989	135	13.7% (11.5% - 15.8%)	<.0001
**NZ European**																
15-64*	224	54	24.1% (16.2% - 32.1%)	232	40	17.2% (12.1% - 22.4%)	222	30	13.5% (9% - 18%)	252	28	11.1% (7.2% - 15%)	251	11	4.4% (1.8% - 6.9%)	<.0001
65-74	236	66	28% (19.8% - 36.1%)	339	52	15.3% (10.8% - 19.9%)	219	28	12.8% (8.4% - 17.2%)	239	27	11.3% (7.3% - 15.3%)	251	16	6.4% (3.3% - 9.4%)	<.0001
75-84	338	114	33.7% (26.6% - 40.9%)	388	90	23.2% (18.6% - 27.8%)	378	61	16.1% (12.4% - 19.9%)	354	65	18.4% (14.3% - 22.4%)	362	41	11.3% (8.1% - 14.6%)	<.0001
85+	130	54	41.5% (29.5% - 53.6%)	169	71	42% (33.8% - 50.2%)	233	86	36.9% (30.7% - 43.1%)	279	92	33% (27.5% - 38.5%)	316	69	21.8% (17.3% - 26.4%)	<.0001
Total	928	288	31% (26.8% - 35.3%)	1128	253	22.4% (19.7% - 25.2%)	1052	205	19.5% (17.1% - 21.9%)	1124	212	18.9% (16.6% - 21.2%)	1180	137	11.6% (9.8% - 13.4%)	<.0001
**Māori**																
15-64*	36	8	22.2% (3% - 41.5%)	48	9	18.8% (7.1% - 30.4%)	53	10	18.9% (8.3% - 29.4%)	74	11	14.9% (6.8% - 23%)	103	7	6.8% (1.9% - 11.7%)	0.0074
65-74	6	4	66.7% (13.1% - 100%)	3	2	66.7% (13.2% - 100%)	22	7	31.8% (12.3% - 51.3%)	22	1	4.5% (0% - 13.3%)	39	7	17.9% (5.9% - 30%)	0.0023
75-84	4	2	50% (0% - 100%)	8	3	37.5% (3.9% - 71.1%)	10	1	10% (0% - 28.6%)	19	5	26.3% (6.5% - 46.1%)	24	9	37.5% (18.1% - 56.9%)	0.9466
85+	0	0	-	2	1	50% (0% - 100%)	4	0	-	2	1	50% (0% - 100%)	5	1	20% (0% - 55.1%)	-
Total	46	14	30.4% (11.6% - 49.3%)	61	15	24.6% (13.1% - 36.1%)	89	18	20.2% (11.9% - 28.6%)	117	18	15.4% (8.8% - 21.9%)	171	24	14% (8.8% - 19.2%)	0.0033
**Pacific**																
15-64*	20	8	40% (9.5% - 70.5%)	51	20	39.2% (24.8% - 53.6%)	66	8	12.1% (4.2% - 20%)	126	12	9.5% (4.4% - 14.7%)	173	17	9.8% (5.4% - 14.3%)	<.0001
65-74	10	4	40% (0% - 83.1%)	21	6	28.6% (7.1% - 50%)	47	8	17% (6.3% - 27.8%)	43	7	16.3% (5.2% - 27.3%)	64	5	7.8% (1.2% - 14.4%)	0.0028
75-84	0	0	-	12	3	25% (0% - 53.3%)	24	6	25% (7.7% - 42.3%)	29	7	24.1% (8.5% - 39.7%)	43	5	11.6% (2% - 21.2%)	-
85+	0	0	-	2	0	-	3	2	66.7% (13.3% - 100%)	7	1	14.3% (0% - 40.2%)	16	4	25% (3.8% - 46.2%)	-
Total	30	12	40% (15.1% - 64.9%)	86	29	33.7% (22.7% - 44.7%)	140	24	17.1% (10.9% - 23.4%)	205	27	13.2% (8.5% - 17.8%)	296	31	10.5% (7% - 14%)	<.0001
**Asian/other**																

15-64*	6	2	33.3% (0% - 86.9%)	15	3	20% (0% - 42.2%)	50	8	16% (5.8% - 26.2%)	76	6	7.9% (1.8% - 14%)	152	13	8.6% (4.1% - 13.0%)	0.0182
65-74	6	2	33.3% (0% - 86.9%)	8	1	12.5% (0% - 37.8%)	42	4	9.5% (0.6% - 18.4%)	58	10	17.2% (7.5% - 27%)	114	7	6.1% (1.7% - 10.6%)	0.0561
75-84	4	4	100% (100% - 100%)	5	1	20% (0% - 55.1%)	21	2	9.5% (0% - 22.1%)	40	10	25% (11.6% - 38.4%)	101	14	13.9% (7.1% - 20.6%)	0.0098
85+	4	2	50% (0% - 100%)	2	0	-	7	2	28.6% (0% - 62.1%)	22	9	40.9% (20.3% - 61.5%)	47	11	23.4% (11.3% - 35.5%)	0.3538
Total	20	10	50% (18.9% - 81.1%)	30	5	16.7% (2.3% - 31.1%)	120	16	13.3% (7.2% - 19.4%)	196	35	17.9% (12.5% - 23.2%)	414	45	10.9% (7.9% - 13.9%)	0.0003

* 16-64 in 2011-2012, § Age-standardised to the WHO world population

N – number of incident strokes (denominator); n – number of all-cause fatal strokes (nominator) within 28 days of the incident stroke onset

Appendix Table 3. 28-day case fatality (%) among first-ever and recurrent stroke cases combined in Auckland, New Zealand in each ARCOS study over the last 40 years (1981-1982, 1991-1992, 2002-2003 and 2011-2012) by age, sex and ethnicity

Age and ethnic group	1981-1982			1991-1992			2002-2003			2011-2012			2021-2022			Trend *P* value
	N	n	% (95% CI)	N	n	% (95% CI)	N	n	% (95% CI)	N	n	% (95% CI)	N	n	% (95% CI)	
**Total**																
15-64*	344	92	26.7% (20.1% - 33.4%)	425	83	19.5% (15.5% - 23.5%)	472	67	14.2% (11% - 17.3%)	611	68	11.1% (8.6% - 13.6%)	784	59	7.5% (5.7% - 9.4%)	<.0001
65-74	366	104	28.4% (21.9% - 35%)	499	89	17.8% (13.9% - 21.7%)	445	66	14.8% (11.5% - 18.1%)	465	59	12.7% (9.7% - 15.7%)	564	43	7.6% (5.4% - 9.8%)	<.0001
75-84	476	168	35.3% (29.2% - 41.4%)	592	146	24.7% (20.8% - 28.5%)	637	135	21.2% (18% - 24.4%)	579	116	20% (16.8% - 23.3%)	679	91	13.4% (10.8% - 16%)	<.0001
85+	174	86	49.4% (38.9% - 60%)	245	103	42% (34.8% - 49.3%)	384	139	36.2% (31.4% - 41%)	441	150	34% (29.6% - 38.4%)	488	114	23.4% (19.6% - 27.1%)	<.0001
Total	1360	450	33.1% (29.5% - 36.6%)	1761	421	23.9% (21.6% - 26.2%)	1938	407	21% (19.2% - 22.8%)	2096	393	18.8% (17.1% - 20.4%)	2515	307	12.2% (10.9% - 13.5%)	<.0001
**Male**																
15-64*	198	48	24.2% (15.8% - 32.7%)	247	40	16.2% (11.3% - 21.1%)	269	35	13% (9% - 17%)	313	30	9.6% (6.3% - 12.8%)	452	28	6.2% (4.0% - 8.4%)	<.0001
65-74	216	58	26.9% (18.5% - 35.2%)	284	53	18.7% (13.2% - 24.1%)	264	36	13.6% (9.5% - 17.8%)	267	34	12.7% (8.7% - 16.7%)	339	20	5.9% (3.4% - 8.4%)	<.0001
75-84	204	62	30.4% (21.4% - 39.3%)	245	61	24.9% (18.9% - 30.9%)	265	50	18.9% (14.2% - 23.6%)	297	59	19.9% (15.3% - 24.4%)	326	43	13.2% (9.5% - 16.9%)	<.0001
85+	44	16	36.4% (16.2% - 56.5%)	41	19	46.3% (31.1% - 61.6%)	94	42	44.7% (34.6% - 54.7%)	135	43	31.9% (24% - 39.7%)	195	45	23.1% (17.2% - 29%)	0.0006
Total	662	184	27.8% (23% - 32.6%)	817	173	21.2% (18% - 24.3%)	892	163	18.3% (15.7% - 20.8%)	1012	166	16.4% (14.1% - 18.7%)	1312	136	10.4% (8.7% - 12%)	<.0001
**Female**																
15-64*	146	44	30.1% (19.6% - 40.7%)	178	43	24.2% (17.5% - 30.8%)	203	32	15.8% (10.7% - 20.8%)	298	38	12.8% (9% - 16.5%)	332	31	9.3% (6.2% - 12.5%)	<.0001
65-74	150	46	30.7% (20.2% - 41.1%)	215	36	16.7% (11.3% - 22.2%)	181	30	16.6% (11.2% - 22%)	198	25	12.6% (8% - 17.3%)	225	23	10.2% (6.3% - 14.2%)	<.0001
75-84	272	106	39% (30.8% - 47.2%)	347	85	24.5% (19.5% - 29.5%)	372	85	22.8% (18.6% - 27.1%)	282	57	20.2% (15.5% - 24.9%)	353	48	13.6% (10% - 17.2%)	<.0001
85+	130	70	53.8% (41.7% - 66%)	204	84	41.2% (33% - 49.3%)	290	97	33.4% (28% - 38.9%)	306	107	35% (29.6% - 40.3%)	293	69	23.5% (18.7% - 28.4%)	<.0001
Total	698	266	38.1% (33% - 43.2%)	944	248	26.3% (23.1% - 29.5%)	1046	244	23.3% (20.8% - 25.9%)	1084	227	20.9% (18.5% - 23.4%)	1203	171	14.2% (12.2% - 16.2%)	<.0001
**NZ European**																
15-64*	272	70	25.7% (18.4% - 33.1%)	287	46	16% (11.5% - 20.5%)	260	33	12.7% (8.6% - 16.7%)	281	28	10% (6.5% - 13.5%)	285	13	4.6% (2.1% - 7%)	<.0001
65-74	340	92	27.1% (20.4% - 33.8%)	446	77	17.3% (13.2% - 21.4%)	292	38	13% (9.2% - 16.9%)	304	34	11.2% (7.6% - 14.7%)	292	17	5.8% (3.1% - 8.5%)	<.0001
75-84	466	162	34.8% (28.6% - 40.9%)	560	137	24.5% (20.5% - 28.4%)	539	109	20.2% (16.8% - 23.6%)	455	83	18.2% (14.7% - 21.8%)	457	54	11.8% (8.9% - 14.8%)	<.0001
85+	170	84	49.4% (38.8% - 60.1%)	239	102	42.7% (35.3% - 50.1%)	340	124	36.5% (31.3% - 41.6%)	394	134	34% (29.3% - 38.7%)	404	90	22.3% (18.2% - 26.3%)	<.0001
Total	1248	408	32.7% (29% - 36.4%)	1532	362	23.6% (21.2% - 26.1%)	1431	304	21.2% (19.1% - 23.4%)	1434	279	19.5% (17.4% - 21.5%)	1438	174	12.1% (10.4% - 13.8%)	<.0001
**Māori**																
15-64*	44	10	22.7% (5.2% - 40.3%)	58	12	20.7% (9.7% - 31.7%)	60	14	23.3% (12.6% - 34%)	85	13	15.3% (7.6% - 23%)	125	9	7.2% (2.7% - 11.7%)	0.0018
65-74	10	6	60% (17% - 100%)	8	3	37.5% (3.9% - 71.1%)	23	7	30.4% (11.6% - 49.3%)	25	2	8% (0% - 18.6%)	51	10	19.6% (8.7% - 30.5%)	0.0049
75-84	6	2	33.3% (0% - 86.8%)	14	4	28.6% (4.9% - 52.3%)	14	3	21.4% (0% - 42.9%)	24	6	25% (7.7% - 42.3%)	30	9	30% (13.6% - 46.4%)	0.9728
85+	0	0	-	2	1	50% (0% - 100%)	5	0	-	4	2	50% (1% - 99%)	6	1	16.7% (0% - 46.5%)	-
Total	60	18	30% (13.6% - 46.4%)	82	20	24.4% (14.7% - 34.1%)	102	24	23.5% (15.3% - 31.8%)	138	23	16.7% (10.4% - 22.9%)	212	29	13.7% (9.1% - 18.3%)	0.0007
**Pacific**																
15-64*	22	10	45.5% (16% - 75%)	63	21	33.3% (21.1% - 45.5%)	85	10	11.8% (4.9% - 18.6%)	151	17	11.3% (6.2% - 16.3%)	203	23	11.3% (7% - 15.7%)	<.0001
65-74	10	4	40% (0% - 83%)	33	7	21.2% (5.9% - 36.5%)	65	14	21.5% (11.5% - 31.5%)	62	12	19.4% (9.5% - 29.2%)	83	9	10.8% (4.1% - 17.5%)	0.0191
75-84	0	0	-	13	4	30.8% (0.9% - 60.6%)	39	12	30.8% (16.3% - 45.3%)	42	10	23.8% (10.9% - 36.7%)	61	8	13.1% (4.6% - 21.6%)	-
85+	0	0	-	2	0	-	8	3	37.5% (3.9% - 71.1%)	15	4	26.7% (4.3% - 49.1%)	20	6	30% (9.9% - 50.1%)	-
Total	32	14	43.8% (19.4% - 68.1%)	111	32	28.8% (19.7% - 38%)	197	39	19.8% (14.2% - 25.4%)	270	43	15.9% (11.6% - 20.3%)	367	46	12.5% (9.1% - 15.9%)	<.0001

**Asian/other**															
15-64*	6	2	33.3% (0% - 86.8%)	17	4	23.5% (1.3% - 45.8%)	64	9	14.1% (5.5% - 22.6%)	94	10	10.6% (4.4% - 16.9%)	171	14	8.2% (4.1% - 12.3%)	0.01
65-74	6	2	33.3% (0% - 86.8%)	12	2	16.7% (0% - 39.8%)	54	5	9.3% (1.5% - 17%)	73	10	13.7% (5.8% - 21.6%)	138	7	5.1% (1.4% - 8.7%)	0.0155
75-84	4	4	100% (100% - 100%)	5	1	20% (0% - 55.1%)	34	8	23.5% (9.3% - 37.8%)	57	17	29.8% (17.9% - 41.7%)	131	20	15.3% (9.1% - 21.4%)	0.0016
85+	4	2	50% (0% - 100%)	2	0	-	10	2	20% (0% - 44.8%)	28	10	35.7% (18% - 53.5%)	58	17	29.3% (17.6% - 41%)	-
Total	20	10	50% (18.9% - 81.1%)	36	7	19.4% (5.5% - 33.4%)	162	24	14.8% (9.3% - 20.3%)	252	47	18.7% (13.8% - 23.5%)	498	58	11.6% (8.8% - 14.5%)	0.0002

* 16-64 in 2011-2012, § Age-standardised to the WHO world population

N – number of first-ever and recurrent stroke cases combined (denominator); n – number of all-cause fatal strokes (nominator) within 28 days of the incident stroke onset

Appendix Figure 1. The overall mean age of individuals with stroke (first-ever and recurrent strokes combined) by ethnicity, 1981-2022

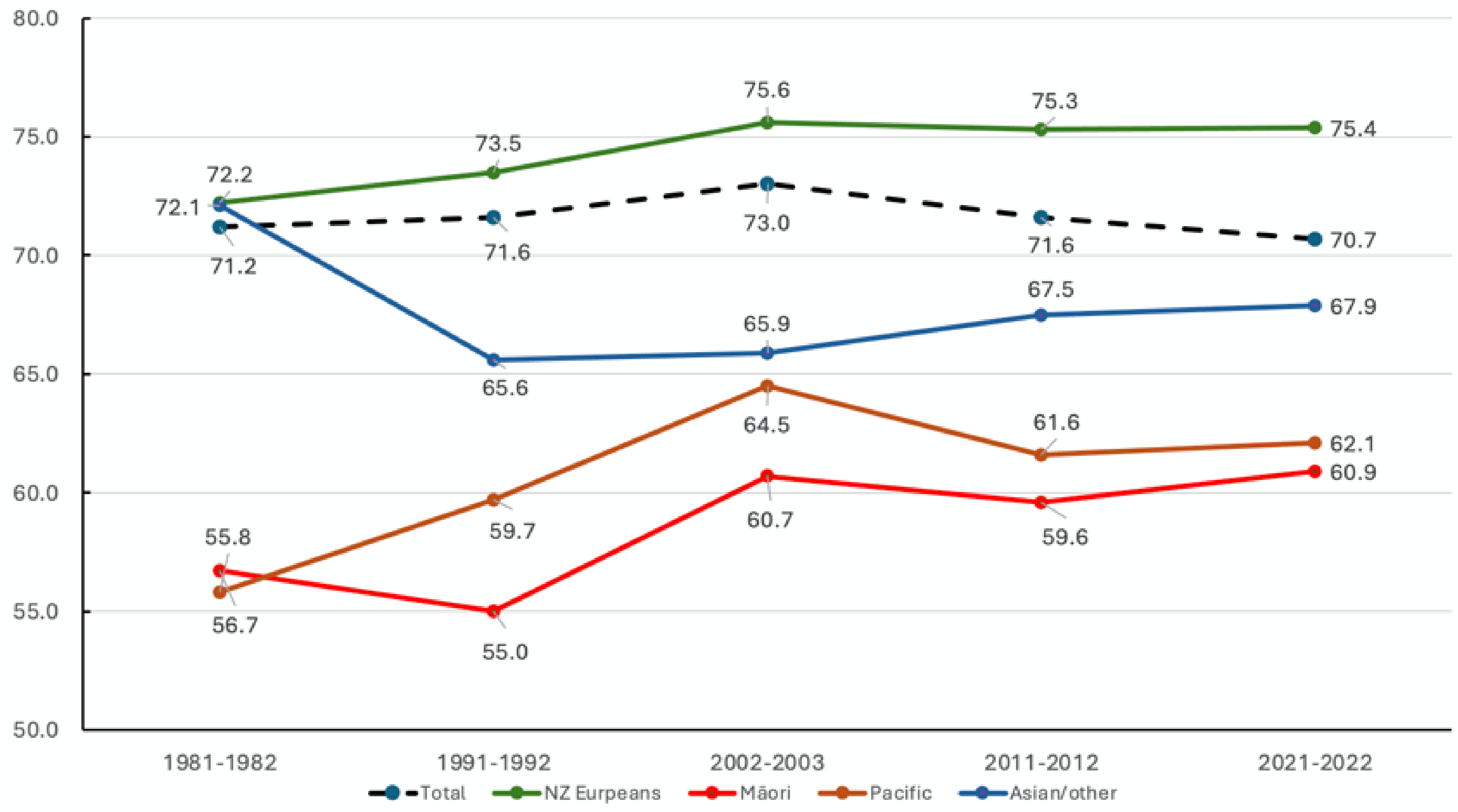

Appendix Figure 2. Trends in the proportions (%) of the pathological types of stroke, 2002-2022

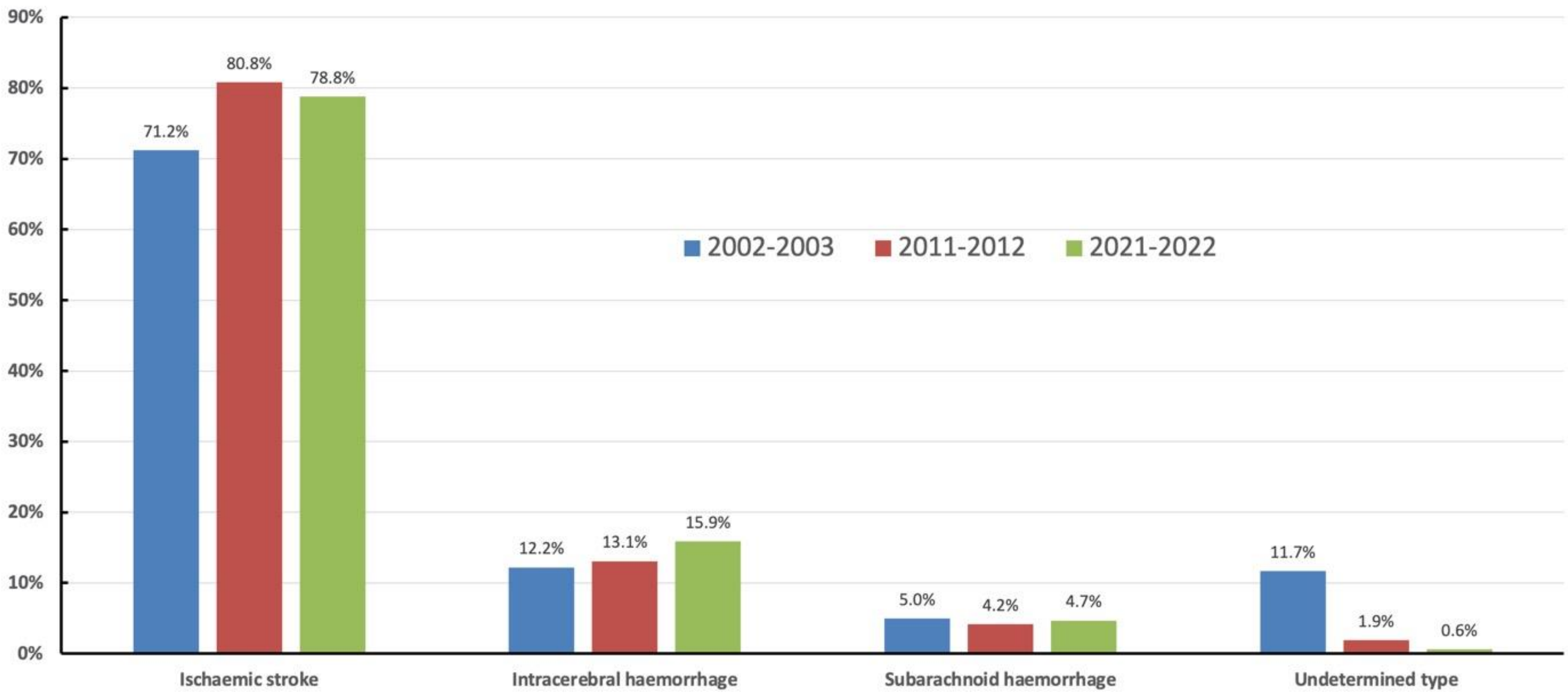

Appendix Figure 3. Age-standardised stroke incidence rates per 100,000/person-years (with 95% CI in upper graph) by ethnicity, 1981-2022

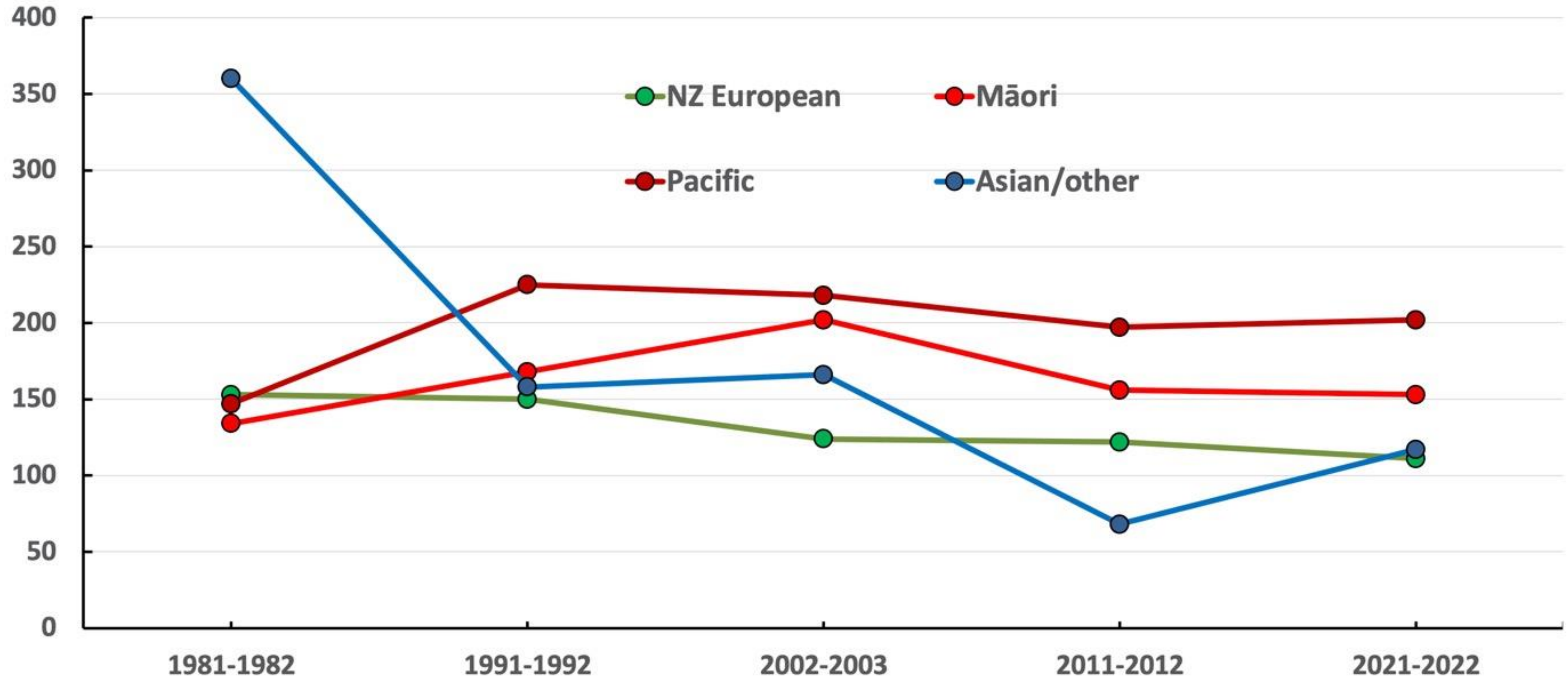

Appendix Figure 4. Trends in (A) age-standardised stroke incidence and attack rates and (B) absolute number of incident and total strokes (incident and recurrent combined), 1981-2022

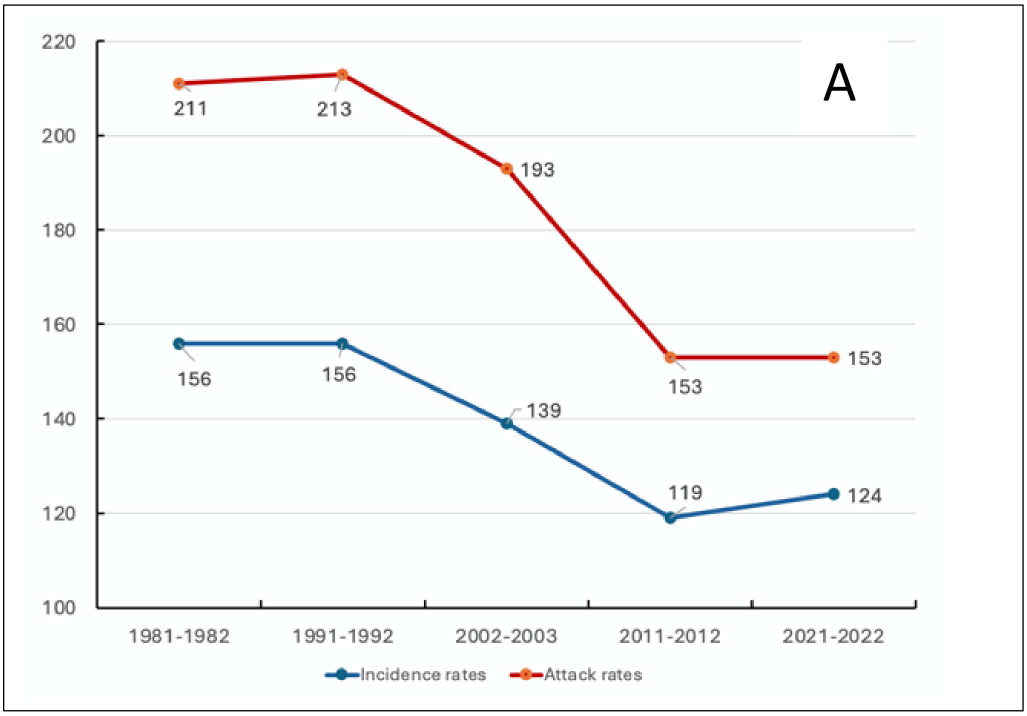

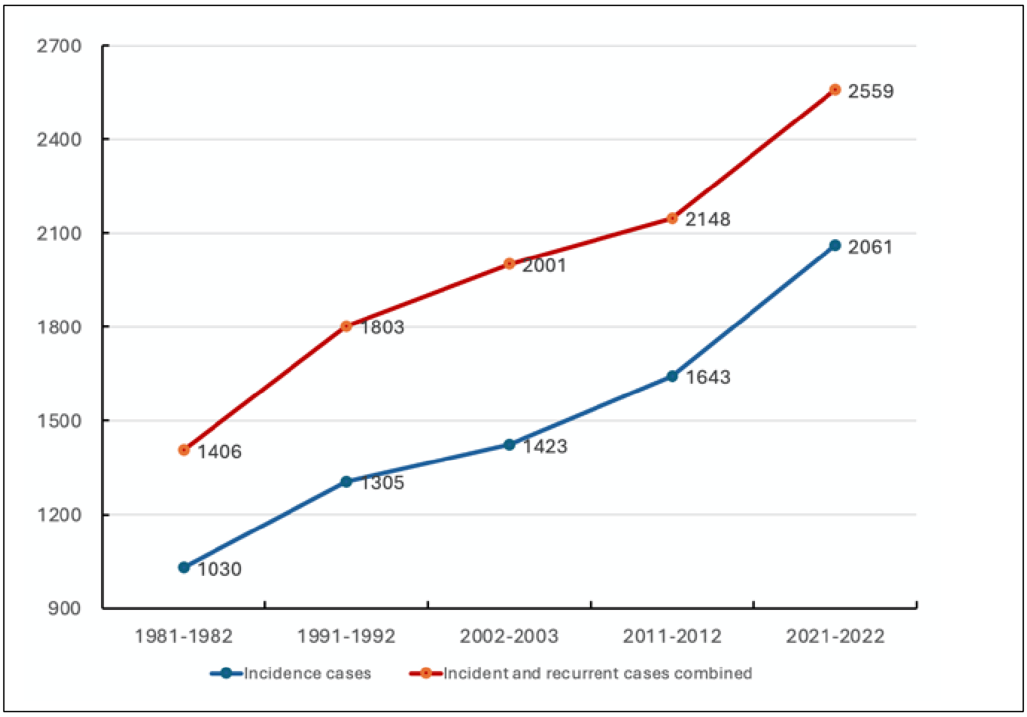

Appendix Figure 5A-C. Forest plots for pooled analysis of average annual percent changes (AAPC) with 95% CIs of A) first-ever stroke incidence in young people, B) first-ever stroke incidence in older people, and C) 1-month case-fatality of first-ever strokes in all ages during the early 21st century

A) Stroke incidence trends of young people (<45 years old in Cincinnati, USA and <55 years old in other studies)

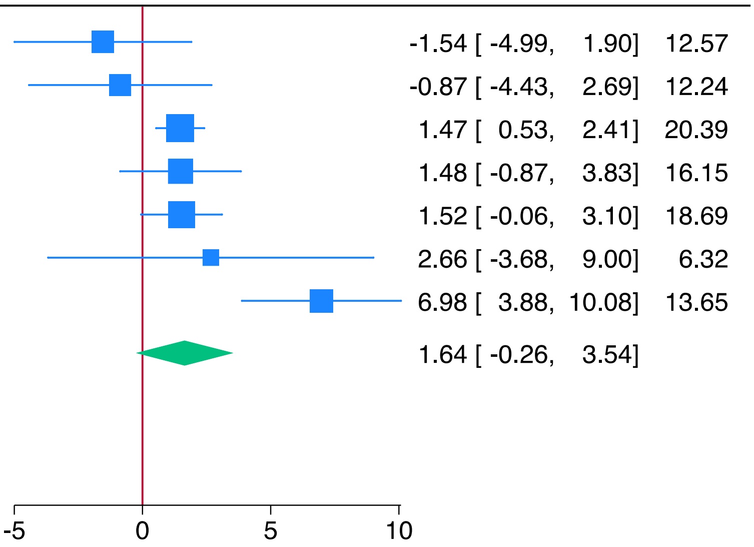

Region (time interval)	AAPC (%) with 95% CI	Weight (%)
Örebro, Sweden (1999 to 2017)	-1.54 [-4.99, 1.90]	12.57
Lund, Sweden (2001-2002 to 2015-2016	-0.87 [-4.43, 2.69]	12.24
Auckland, New Zealand (2002-2003 to 2021-2022)	1.47 [0.53, 2.41]	20.39
Martinique (1999 to 2012)	1.48 [-0.87, 3.83]	16.15
Cincinnati, USA (1999 to 2015)	1.52 [-0.06, 3.10]	18.69
Arcadia, Greece (2004 to 2015-2016)	2.66 [-3.68, 9.00]	6.32
Oxfordshire, UK (2002-2010 to 2010-2018)	6.98 [3.88, 10.08]	13.65
**Overall**	1.64 [-0.26, 3.54]	

Heterogeneity: $\tau^2 = 4.37$, $I^2 = 77.91\%$, $H^2 = 4.53$

Test of $\theta_i = \theta_j$: $Q(6) = 16.80$, $p = 0.01$

Test of $\theta = 0$: $z = 1.70$, $p = 0.09$

Random-effects REML model

B) Stroke incidence trends of older people (≥45 years old in Cincinnati, USA and ≥55 years old in other studies)

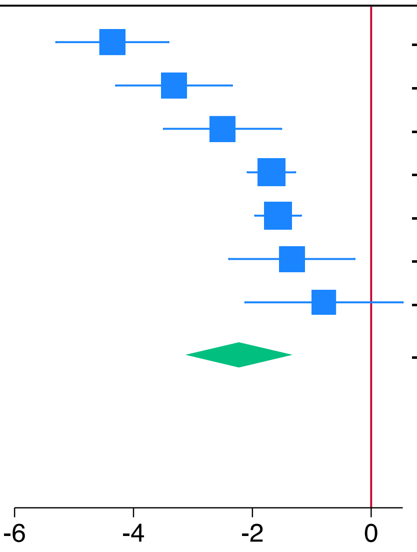

Region (time interval)	AAPC (%) with 95% CI	Weight (%)
Örebro, Sweden (1999 to 2017)	-4.36 [-5.30, -3.41]	14.08
Martinique (1999 to 2012)	-3.32 [-4.29, -2.34]	13.93
Lund, Sweden (2001-2002 to 2015-2016	-2.50 [-3.49, -1.51]	13.87
Cincinnati, USA (1999 to 2015)	-1.68 [-2.08, -1.28]	16.15
Auckland, New Zealand (2002-2003 to 2021-2022)	-1.57 [-1.95, -1.18]	16.19
Oxfordshire, UK (2002-2010 to 2010-2018)	-1.34 [-2.39, -0.28]	13.55
Arcadia, Greece (2004 to 2015-2016)	-0.80 [-2.12, 0.53]	12.23
**Overall**	-2.23 [-3.12, -1.33]	

Heterogeneity: $\tau^2 = 1.25$, $I^2 = 91.01\%$, $H^2 = 11.12$

Test of $\theta_i = \theta_j$: $Q(6) = 43.38$, $p = 0.00$

Test of $\theta = 0$: $z = -4.87$, $p = 0.00$

Random-effects REML model

C) Stroke case-fatality trends of all ages

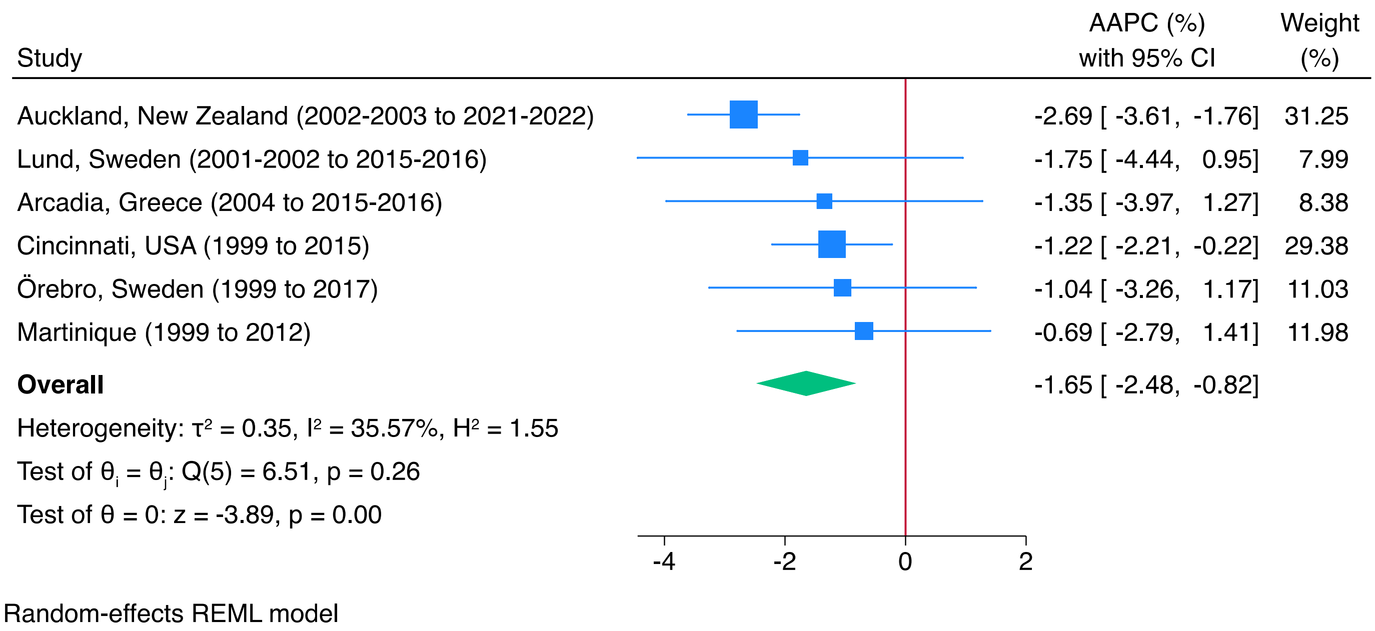

References

1. Aho K, Harmsen P, Hatano S, Marquardsen J, Smirnov VE, Strasser T. Cerebrovascular disease in the community: results of a WHO collaborative study. *Bulletin of the World Health Organization*. 1980;58:113-130.
2. International Classification of Diseases, Eleventh Revision (ICD-11), World Health Organization (WHO) 2019/2021 https://icd.who.int/browse11 Accessed 26 March 2024.
3. Bonita R, Broad JB, Beaglehole R. Changes in stroke incidence and case-fatality in Auckland, New Zealand, 1981-91. *Lancet*. 1993;342:1470-1473.
4. Nichols EK, Byass P, Chandramohan D, Clark SJ, Flaxman AD, Jakob R, Leitao J, Maire N, Rao C, Riley I, et al. The WHO 2016 verbal autopsy instrument: An international standard suitable for automated analysis by InterVA, InSilicoVA, and Tariff 2.0. *PLoS Med*. 2018;15:e1002486. doi: 10.1371/journal.pmed.1002486
5. Bonita R, Broad JB, Beaglehole R. Ethnic differences in stroke incidence and case fatality in Auckland, New Zealand. *Stroke*. 1997;28:758-761.
6. Feigin VL, Krishnamurthi RV, Barker-Collo S, McPherson KM, Barber PA, Parag V, Arroll B, Bennett DA, Tobias M, Jones A, et al. 30-year trends in stroke rates and outcome in Auckland, New Zealand (1981-2012): a multi-ethnic population-based series of studies. *PLoS One*. 2015;10:e0134609. doi: 10.1371/journal.pone.0134609
7. Anderson CS, Carter KN, Hackett ML, Feigin V, Barber PA, Broad JB, Bonita R. Trends in stroke incidence in Auckland, New Zealand, during 1981 to 2003. *Stroke*. 2005;36:2087-2093.
8. Krishnamurthi R., Jones A., Barber A., Barker-Collo S., McPherson K., Bennett D., Rush E., Suh F., Starkey N., Theadom A., et al. Methodology of a Population-Based Stroke and TIA Incidence and Outcomes Study: The Auckland Regional Community Stroke Study (ARCOS IV) 2011-2012. *International Journal of Stroke*. 2014;9:140-147.
9. Bonita R, Beaglehole R, North JD. Subarachnoid hemorrhage in New Zealand: an epidemiological study. *Stroke*. 1983;14:342-347.
10. Adams HP, Jr., Bendixen BH, Kappelle LJ, Biller J, Love BB, Gordon DL, Marsh EE, III. Classification of subtype of acute ischemic stroke. Definitions for use in a multicenter clinical trial. TOAST. Trial of Org 10172 in Acute Stroke Treatment. *Stroke*. 1993;24:35-41.
11. Rankin J. Cerebral vascular accidents in patients over the age of 60: II. Prognosis. *ScottishMedicalJournal*. 1957;2:200-213.
12. Hook EB, Regal RR. Capture-Recapture Methods in Epidemiology: Methods and Limitations. *Epidemiologic Reviews*. 1995;17:243-264. doi: 10.1093/oxfordjournals.epirev.a036192
13. Tilling K, Sterne JA, Wolfe CD. Estimation of the incidence of stroke using a capture-recapture model including covariates. *IntJ Epidemiol*. 2001;30:1351-1359.
14. Scott CA, Li L, Rothwell PM. Diverging Temporal Trends in Stroke Incidence in Younger vs Older People: A Systematic Review and Meta-analysis. *JAMA Neurology*. 2022;79:1036-1048. doi: 10.1001/jamaneurol.2022.1520
15. Page MJ, McKenzie JE, Bossuyt PM, Boutron I, Hoffmann TC, Mulrow CD, Shamseer L, Tetzlaff JM, Akl EA, Brennan SE, et al. The PRISMA 2020 statement: An updated guideline for reporting systematic reviews. *J Clin Epidemiol*. 2021;134:178-189. doi: 10.1016/j.jclinepi.2021.03.001
16. New Zealand Net Migration Rate 1950-2024. https://www.macrotrends.net/global-metrics/countries/NZL/new-zealand/net-migration Accessed 24 December 2024.
